# Cell type–specific mechanism of Setd1a heterozygosity in schizophrenia pathogenesis

**DOI:** 10.1126/sciadv.abm1077

**Published:** 2022-03-04

**Authors:** Renchao Chen, Yiqiong Liu, Mohamed N. Djekidel, Wenqiang Chen, Aritra Bhattacherjee, Zhiyuan Chen, Ed Scolnick, Yi Zhang

**Affiliations:** 1 Howard Hughes Medical Institute, Boston Children’s Hospital, Boston, MA 02115, USA.; 2 Program in Cellular and Molecular Medicine, Boston Children’s Hospital, Boston, MA 02115, USA.; 3 Division of Hematology/Oncology, Department of Pediatrics, Boston Children’s Hospital, Boston, MA 02115, USA.; 4 The Stanley Center for Psychiatric Research at Broad Institute, Cambridge, MA 02142, USA.; 5 Department of Genetics, Harvard Medical School, Boston, MA 02115, USA.; 6 Harvard Stem Cell Institute, WAB-149G, 200 Longwood Avenue, Boston, MA 02115, USA.

## Abstract

Schizophrenia (SCZ) is a chronic, serious mental disorder. Although more than 200 SCZ-associated genes have been identified, the underlying molecular and cellular mechanisms remain largely unknown. Here, we generated a Setd1a (SET domain containing 1A) haploinsufficiency mouse model to understand how this SCZ-associated epigenetic factor affects gene expression in brain regions highly relevant to SCZ. Single-cell RNA sequencing revealed that Setd1a heterozygosity causes highly variable transcriptional adaptations across different cell types in prefrontal cortex (PFC) and striatum. The *Foxp2*^+^ neurons exhibit the most prominent gene expression changes among the different neuron subtypes in PFC, which correlate with changes in histone H3 lysine 4 trimethylation. Many of the genes dysregulated in Setd1a^+/−^ mice are involved in neuron morphogenesis and synaptic function. Consistently, Setd1a^+/−^ mice exhibit certain behavioral features of patients with SCZ. Collectively, our study establishes Setd1a^+/−^ mice as a model for understanding SCZ and uncovers a complex brain region– and cell type–specific dysregulation that potentially underlies SCZ pathogenesis.

## Teaser

Haploinsufficiency of schizophrenia-associated gene Setd1a causes cell type-specific transcriptional changes in the brain.

## INTRODUCTION

Schizophrenia (SCZ) is a chronic and severe mental disorder often emerging in young adulthood, which affects ~1% of the total population (*1*). Previous studies have revealed that genetic factors play a major role in the etiology of SCZ. For example, twin studies have estimated that the heritable liability to SCZ was about 80% (*2*). During the past decade, large-scale genome-wide association study (GWAS) and exome sequencing studies have identified more than 200 SCZ-associated loci (*3*–*7*). However, the mechanism underlying how genetic perturbation leads to SCZ pathophysiology remains largely elusive, which prevents mechanism-based drug discovery. Recent studies integrating SCZ-associated loci with cell type–specific transcriptomes of single-cell RNA sequencing (scRNA-seq) have suggested that different neuron types in certain brain regions have distinct roles in SCZ (*8*, *9*), emphasizing the needs of understanding cell type–specific contribution to the pathophysiology of SCZ.

Epigenetic regulation plays an important role in the development and function of the nervous system, and accumulating evidence has linked epigenetic dysregulation with psychiatric disorders, including SCZ (*10*, *11*). For example, analyses of large GWAS dataset has identified histone methylation as a biological pathway showing the strongest association with psychiatric disorders, including SCZ (*12*). In addition, decreased histone H3 lysine 4 trimethylation (H3K4me3) in promoter regions and down-regulation of corresponding genes involved in inhibitory neural transmission (such as *GAD1*) has been observed in patients with SCZ (*13*). Furthermore, epigenetic profiling with neuronal and non-neuronal cells from cortical samples of patients with SCZ revealed that SCZ risk variants are enriched in neuronal H3K4me3 landscapes (*14*). Consistent with these findings, several recent studies, including parent-proband trio study (*15*) focusing on de novo mutations and case-controlled large-scale exome sequencing (*16*), have identified loss-of-function mutations on human *SETD1A* in patients with SCZ, suggesting that haploinsufficiency of *SETD1A* contributes to the pathogenesis of SCZ. As an H3K4 methyltransferase, mouse Setd1a plays an important role in regulating transcription (*17*), and its homozygous loss of function leads to early embryonic lethality (*18*). However, the function of Setd1a in the nervous system and how its heterozygosity contributes to pathophysiology of SCZ are unknown. As an epigenetic regulator and a confirmed SCZ risk gene, modeling *Setd1a* loss of function provides a unique opportunity for understanding epigenetic-based transcriptional mechanism of SCZ pathogenesis.

Similar to many other psychotic disorders, patients with SCZ show highly heterogenous neurological symptoms, and, in general, it is difficult to link specific genetic mutations to particular symptoms. Despite these difficulties, recent studies using SCZ animal models have begun to reveal brain region–, cell type–, and developmental stage–specific pathologic mechanism of SCZ (*19*–*21*), suggesting that different brain region/cell types are not uniformly affect by a specific genetic variation. Thus, dissecting the function of SCZ-associated genes, especially their brain region/cell type–specific function, is critical for understanding the complex pathophysiology and improving SCZ treatment.

Here, we generated a *Setd1a* heterozygous mouse model and performed single-cell transcriptome profiling to assess gene expression changes across different cell types in prefrontal cortex (PFC) and striatum, two brain regions tightly associated with SCZ. Comparative transcriptome analyses not only revealed cell type– and brain region–specific transcriptional maladaptation but also uncovered neuron subtypes with most prominent changes in both PFC and striatum in response to *Setd1a* heterozygosity. By further performing cell type–specific transcriptional and epigenetic profiling in the *Foxp2*^+^ neurons, the subtype in PFC with the strongest gene expression changes, we found that Setd1a deficiency cause H3K4me3 decrease in thousands of genomic loci, which likely underlie the transcriptional changes in these neurons. Notably, both scRNA-seq and cell type–specific profiling revealed transcriptional dysregulation of genes associated with SCZ and are involved in regulating neuron morphogenesis and synaptic function. Collectively, our study suggests that Setd1a deficiency–induced H3K4me3 decrease underlies the cell type–specific transcriptional changes in *Setd1a*^+/−^ mice, which causes the cellular and behavioral dysfunctions of this SCZ mouse model.

## RESULTS

### Generation of a *Setd1a* heterozygous loss-of-function mouse model

Previous studies have identified *SETD1A* as one of the mutated genes in patients with SCZ (*15*, *16*). Although the location of the mutations on *SETD1A* gene differ from one another in different patients, all of them cause premature translation termination before the SET [Su(var)3-9, Enhancer-of-zeste and Trithorax] domain of the SETD1A protein, resulting in loss of methyltransferase activity. To mimic these loss-of-function mutations in a mouse model, we used CRISPR-Cas9 technique to target the 15th exon of *Setd1a* (fig. S1A), which is right before the SET domain encoded by exons 16 to 18. By screening the Cas9-targeted offsprings, we identified an 8–base pair (bp)–deleted mutation that causes frameshift and premature translation termination (fig. S1A), mimicking the human *SETD1A* loss-of-function mutations in patients with SCZ. The mutated DNA sequences were confirmed by target DNA sequencing (fig. S1A), and the decrease in Setd1a protein level in heterozygous *Setd1a* mice was confirmed by Western blot analysis (fig. S1B).

### Cell type–specific transcriptional changes in Setd1a^+/−^ mouse PFC

Setd1a is a histone methyltransferase involved in transcriptional activation by depositing the active histone mark H3K4me3 in the genome (*17*). However, its function in the nervous system and how its dysfunction is linked to SCZ remain elusive. We hypothesize that *Setd1a* heterozygosity could cause aberrant gene expression by affecting H3K4 methylation status, which contributes to SCZ pathology. To test this hypothesis, we performed comparative transcriptome analyses between control and *Setd1a*^+/−^ mouse PFC and striatum, two brain regions strongly implicated in SCZ pathogenesis. To overcome the high cellular heterogeneity issue of these brain regions, we used scRNA-seq, aiming to reveal brain region– and cell type–specific transcriptional effect of *Setd1a* haploinsufficiency.

**Fig. 1.**
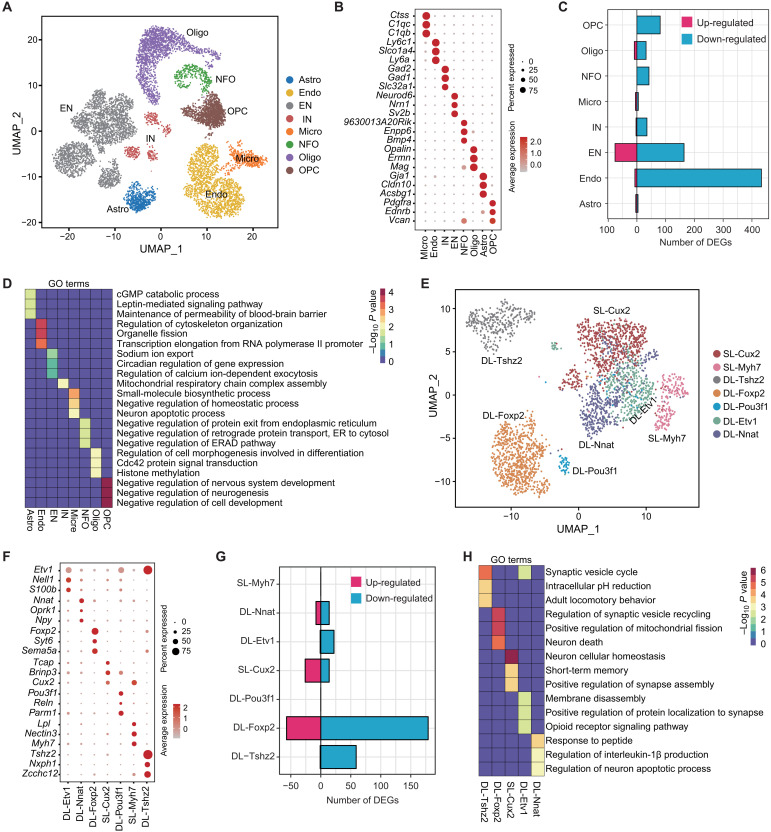
Cell type–specific transcriptional changes in the PFC of *Setd1a*^+/−^ mice. (**A**) Uniform manifold approximation and projection (UMAP) plot showing the major cell types in the PFC. Astro, astrocyte; Endo, endothelial cell; EN, excitatory neuron; IN, interneuron; Micro, microglia; NFO, newly formed oligodendrocyte; Oligo, oligodendrocyte; OPC, oligodendrocyte progenitor cell; ER, endoplasmic reticulum; ERAD, endoplasmic-reticulum-associated protein degradation. (**B**) Dot plot showing the expression of selective markers in different PFC cell types. (**C**) Bar graph showing the number of differentially expressed genes (DEGs) in different cell populations of PFC in *Setd1a*^+/−^ mice. (**D**) Heatmap showing the Gene Ontology (GO) terms enriched for the DEGs of different cell types in PFC. The −log_10_(*P* value) of GO terms across different cell types are color-coded. cGMP, cyclic guanosine 3′,5′-monophosphate. (**E**) UMAP plot showing the different excitatory neuron subtypes in the PFC. DL, deep layer subtype; SL, superficial layer subtype. (**F**) Dot plot showing the expression of marker genes in different excitatory neuron subtypes of PFC. (**G**) Bar graph showing the number of DEGs across excitatory neuron subtypes in PFC. (**H**) Heatmap showing the GO terms enriched for the DEGs of different excitatory neuron subtypes in PFC. The −log_10_(*P* value) of GO terms in different neuron subtypes are color-coded.

To this end, we dissected PFC tissues from acute coronal sections of Setd1a^+/−^ and wild-type (WT) mouse brains and dissociated the tissues to single cells before processing with the 10x Chromium platform (10x Genomics). We profiled two biological replicates for each genotype, with each replicate with cells from two animals. For clustering analysis, we combined cells from WT and *Setd1a*^+/−^ animals using canonical cross-correlation (5303 cells from WT mice and 3694 cells from *Setd1a*^+/−^ mice), which revealed eight transcriptionally distinct cell clusters (Fig. 1A). On the basis of the expression of known marker genes, we annotated these clusters as excitatory neuron, inhibitory neuron, and glia cell types (Fig. 1B), which cover all known major cell types in PFC. We observed no significant changes in the proportion of these different cell types between WT and *Setd1a*^+/−^ mice (fig. S1, C and D), indicating that *Setd1a* haploinsufficiency does not alter cell composition in PFC.

To identify *Setd1a* heterozygosity–induced changes on gene expression in a cell type–specific context, we compared transcriptional profiles between the cells from WT and *Setd1a*^+/−^ groups [with cutoff fold change (FC) > 1.5 and a false discovery rate (FDR) < 0.05] within each of the eight cell types. The results showed that *Setd1a* heterozygosity resulted in broad but distinct changes across different cell types, with endothelial cells and excitatory neurons exhibiting the highest number of differentially expressed genes (DEGs) (Fig. 1C and table S1), suggesting that they are the most affected cell types by *Setd1a* deficiency. In addition, inhibitory neurons and oligodendrocyte showed moderate transcriptional alterations, while astrocyte and microglia showed little changes (Fig. 1C). Notably, we did not find strong correlation between the number of DEGs and the abundance of the cell types, as cell types with comparable abundance exhibit marked difference in their transcriptional changes (e.g., endothelial cells versus oligodendrocytes and newly formed oligodendrocytes versus microglia). Furthermore, most DEGs in different PFC cell types are down-regulated in *Setd1a*^+/−^ mice (Fig. 1C), consistent with the established function of Setd1a in transcriptional activation. Only a small number of DEGs are shared between different cell types, suggesting that *Setd1a* deficiency leads to broad but largely cell type–specific transcriptional changes in PFC (fig. S1E).

To gain insights into the functional consequence of the transcriptional maladaptation of PFC, we performed Gene Ontology (GO) analysis on DEGs of different cell types. Consistent with the observation that DEGs are largely cell type specific, we found enrichment of distinct GO terms across different cell types (Fig. 1D). In the excitatory neurons, the DEGs are enriched in terms like sodium ion export and regulation of calcium ion–dependent exocytosis (Fig. 1D). Consistently, similar terms were enriched in ingenuity pathway analysis (IPA), such as influx of Ca^2+^, transport of synaptic vesicles, and neurotransmission (fig. S1F), suggesting that Setd1a insufficiency leads to dysfunction of synaptic vesicle exocytosis in these neurons. Furthermore, we found terms like SCZ spectrum disorder and SCZ among the top terms in the IPA “Disease and Bio Functions” analysis (fig. S1F), and a direct search of the DEGs revealed that several of them are SCZ-associated genes identified in human GWAS studies (fig. S1G and table S2), such as *Taok2*, *Scaf1*, and *Nsun6*. In addition, using a bootstrapped test, we found that SCZ-associated genes were significantly overlapping with the DEGs of excitatory neurons (fig. S1G). However, using MAGMA (multi-marker analysis of genomic annotation), a more rigorous method that takes confounding factor such as gene size and linkage disequilibrium into account (*8*), we did not find significant enrichment of SCZ-associated genes in the DEGs of different cell types. In addition to excitatory neurons, marked transcriptional changes have been observed in the endothelial cells of PFC (Fig. 1C). GO and IPA analysis of these DEGs revealed both common and distinct terms. For example, both GO and IPA showed that DEGs are enriched in organization of cytoskeleton (such as *Lima1*, *Tiam1*, and *Arhgdia*) and transcriptional regulation (such as *Jun*, *Chd3*, and *Stat2*) (Fig. 1D and fig. S1H). In addition, GO analysis revealed terms like organelle fission, which were not found in IPA, while IPA also revealed specific terms like apoptosis (such as *Fastk*, *Cd2ap*, and *Ifnar1*) (Fig. 1D and fig. S1H). In addition, terms specifically related to the vascular system, such as vasculogenesis and abnormal morphology of cardiovascular system (such as *Pecam1*, *Sema4d*, and *Vezf1*), were also specifically identified by IPA (fig. S1H). Notably, we found SCZ-associated genes (such as *Epn2*, *Stag1*, and *Lima1*) were significantly enriched in DEGs of endothelial cells (fig. S1G and table S2), supporting the notion that the malfunctioning of the vascular system in the *Setd1a*^+/−^ could also play a role in SCZ-related dysfunction.

### Neuron subtype–specific transcriptional changes in *Setd1a*^+/−^ mouse PFC

It has been well established that the cortex is organized into different layers that have distinct neuronal composition, connectivity, and function (*22*). To further determine whether *Setd1a* heterozygosity results in similar or distinct changes among different cortical neuron subtypes, we focused our analysis on excitatory neuron, the most abundant neuron type in PFC. Unbiased classification of the excitatory neuron population revealed seven subtypes (Fig. 1, E and F, and fig. S1I). Consistent with the layer structure of PFC, markers of different excitatory neuron subtypes are enriched in different cortical layers (fig. S1J), which allowed us to annotate these subtypes by combining their spatial information (superficial or deep cortical layer) and marker gene expression (Fig. 1, E and F).

Although the relative abundance of these excitatory neuron subtypes is not affected by *Setd1a* heterozygosity (fig. S1K), the number of DEGs in each neuron subtypes varies greatly with the deep layer (DL)–Foxp2 subtype (Foxp2-expressing neurons located in the deep cortical layer VI) affected the most (Fig. 1G and table S3). Consistent with the general trends of transcriptional repression in the whole excitatory neuron population, most DEGs identified from excitatory neuron subtypes were down-regulated in *Setd1a*^+/−^ mice (Fig. 1G). Although some excitatory neuron subtypes [such as superficial layer (SL)–Myh7 and DL-Pou3f1] with very few DEGs identified might be a result of their small cluster size (Fig. 1E), the DL-Etv1 and SL-Cux2 subtypes with comparable cell numbers to that of the DL-Foxp2 subtype also showed much fewer DEGs (Fig. 1G), indicating that *Setd1a* heterozygosity–caused transcriptional changes varied substantially among different excitatory neuron subtypes. Few DEGs were shared among the different PFC excitatory neuron subtypes (fig. S1L), indicating that *Setd1a* heterozygosity–caused transcriptional changes are largely neuron subtype specific. Notably, we found that several SCZ-related genes, including *Arl2*, *Ppdpf*, *Kit*, and *Taok2*, were specifically dysregulated in the DL-Foxp2 subtype in the *Setd1a*^+/−^ PFC, suggesting that transcriptional maladaptation in this neuron subtype might be particularly relevant to the SCZ pathology.

To further explore potential cell type–specific functional changes, we performed pathway analysis on DEGs in different neuron subtypes. Although few DEGs overlap among different neuron subtypes, GO analysis revealed that the terms related to synaptic function are enriched in multiple neuron subtypes (Fig. 1H). For example, we found that DEGs in DL-Tshz1, DL-Foxp2, SL-Cux2, and DL-ETV1 subtypes were enriched in terms like synaptic vesicle recycle, regulation of synaptic vesicle recycling, positive regulation of synapse assembly, and positive regulation of protein localization to synapse, respectively (Fig. 1H), indicating that divergent transcriptional changes in different neuron subtypes may converge on common pathway that impairs synaptic function across PFC excitatory neurons. On the other hand, subtype-specific GO terms related to neural function were also enriched in different excitatory neuron subtypes, such as intracellular pH reduction (DL-Tshz2), positive regulation of mitochondrial fission (DL-Foxp2), neuron cellular homeostasis (SL-Cux2), and opioid receptor signal pathway (DL-Etv1) (Fig. 1H), indicating that *Setd1a* heterozygosity also affects neuronal functions in neuron subtype–specific manner. Collectively, single-cell transcriptomic comparison of PFC tissue from WT and *Setd1a*^+/−^ animals not only revealed cell type and neuron subtypes-specific transcriptional changes but also uncovered convergent and divergent neural mechanisms that may underlie functional impairments of *Setd1a*^+/−^ mice.

### Cell type–specific transcriptional changes in *Setd1a*^+/−^ mouse striatum

**Fig. 2.**
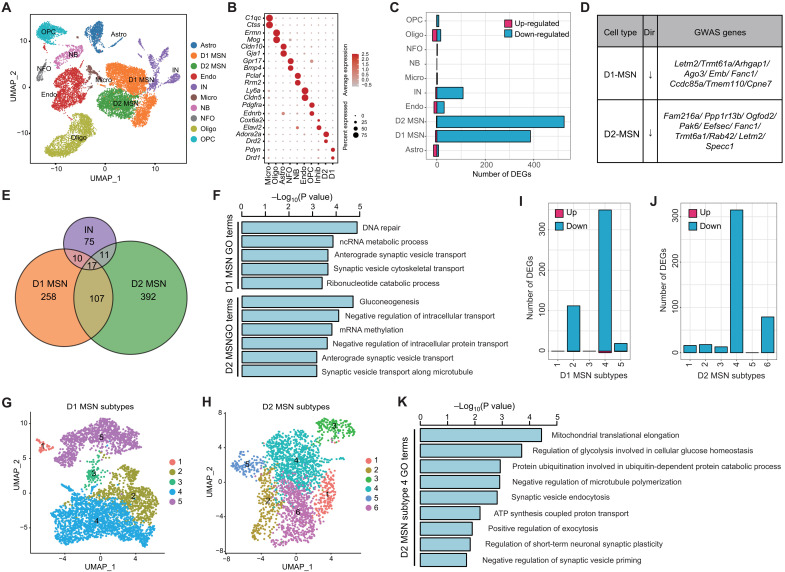
Cell type–specific transcriptional changes in the striatum of *Setd1a*^+/−^ mice. (**A**) UMAP plot showing the major cell types in striatum. D1 MSN, D1 dopamine receptor–expressing MSN; D2 MSN, D2 dopamine receptor–expressing MSN; NB, neural stem cells and neuroblast. (**B**) Dot plot showing the expression of selected marker genes in striatal cell types. (**C**) The number of DEGs in different striatal cell populations of *Setd1a*^+/−^ mice. (**D**) DEGs in D1 and D2 MSNs that are also identified as SCZ-associated genes by GWAS studies. (**E**) Overlap of DEGs between WT and *Setd1a*^+/−^ mice in D1 MSN, D2 MSN, and interneuron. (**F**) Bar graph showing the GO terms enriched in the DEGs in D1 and D2 MSNs. (**G** and **H**) UMAP plots showing the D1 (G) and D2 (H) MSN subtypes in striatum. (**I** and **J**) The number of DEGs in D1 (I) and D2 (J) MSN subtypes of striatum in *Setd1a*^+/−^ mice. (**K**) Bar graph showing GO terms enriched in the DEGs of D2 MSN subtype 4.

In addition to PFC, striatum is another brain region tightly associated with SCZ. Thus, we performed similar scRNA-seq to analyze the transcriptional effect of *Setd1a* heterozygosity in striatum. After quality control, we obtained transcriptome profiles of 14,690 high-quality single cells dissociated from striatum (include both dorsal and ventral striatum) of WT and *Setd1a*^+/−^ mice (8400 cells from WT mice and 6290 cells from *Setd1a*^+/−^ mice). Clustering analysis of these cells revealed 10 major cell clusters (Fig. 2A). On the basis of cluster-specific markers (Fig. 2B), the clusters represent different cell types, including D1 and D2 medium spiny neurons (MSNs), an interneuron population and a newborn neuron population, as well as other non-neuron cell types (Fig. 2, A and B). Similar to the observation in PFC, the relative abundance of different cell types was not changed by Setd1a heterozygosity (fig. S2, A and B), indicating that the striatal cell composition was not altered in the *Setd1a*^+/−^ mice.

Comparative transcriptome analysis between cells from WT and *Setd1a*^+/−^ animals within each of the 10 cell types revealed cell type–specific DEGs in striatum (FC > 2 and FDR < 0.05), with most of which down-regulated in *Setd1a*^+/−^ animals (Fig. 2C and table S4). Similar to PFC, different striatal cell types exhibited great variation in the number of DEGs. Among the four neuronal populations, D1 and D2 MSNs showed the most marked gene expression changes, with 392 and 527 DEGs detected in these two neuron types, respectively (Fig. 2C). In addition, SCZ-associated genes identified by GWAS are present in the DEGs of D1 and D2 MSNs, but bootstrapped overlap test or MAGMA did not find significant overlap (Fig. 2D and fig. S2C). Only a few SCZ-associated genes are altered in both D1 and D2 MSNs (*Letm2*, *Trmt6a1*, and *Fanc1*), while most of them only changed in either D1 (e.g., *Arhgap1*, *Emb*, and *Cpne7*) or D2 MSNs (e.g., *Ppp1r13b*, *Rab42*, and *Pak6*) (Fig. 2D). In general, D2 MSNs exhibited more DEGs than D1 MSNs, indicating that D2 MSN is more severely affected than D1 MSN in the striatum of *Setd1a*^+/−^ mice, which is consistent with the fact that many antipsychotic drugs for treating patients with SCZ are D2 dopamine receptor antagonists (*23*). In interneuron, we found 113 genes showed significant changes on their mRNA level (Fig. 2C). Although previous studies have reported defective neurogenesis in patients with SCZ and an animal model (*24*), little transcriptional changes in newborn neurons of *Setd1a*^+/−^ mice were observed (Fig. 2C), suggesting that neurogenesis in the lateral ventricle wall is not affected by *Setd1a* heterozygosity. Compared to the neuronal cells, non-neuronal cell types in striatum show much milder transcriptional changes (Fig. 2C), indicating that *Setd1a* heterozygosity has a stronger impact on neurons than non-neuronal cells in striatum. We further assessed the overlap of DEGs from D1 MSN, D2 MSN, and interneuron and found that most transcriptional changes are neuron type specific, with only limited number of DEGs that are shared between the different striatal neuron types (Fig. 2E), indicating that *Sed1a* heterozygosity largely causes neuron type–specific transcriptional changes in striatum.

To gain insights into how the transcriptional changes may affect the neuronal functions, we performed GO analysis with DEGs identified in D1 MSN, D2 MSN, and interneurons. Consistent with the minor overlap of DEGs among the different neuron populations, largely distinct biological terms were found in different neuron types. For example, DNA repair and noncoding RNA (ncRNA) metabolic process were enriched in DEGs in the D1 MSN, gluconeogenesis and RNA methylation were enriched in DEGs in the D2 MSN, and regulation of neurological system process and long-term memory were enriched in DEGs in inhibitory neurons (Fig. 2F and fig. S2D). On the other hand, terms related to synaptic function, such as anterograde synaptic vesicle transport, were shared between D1 and D2 MSNs (Fig. 2F). Considering that synapsis-related GO terms were also identified in DEGs of PFC excitatory neurons (Fig. 1H), impairment of synaptic function is likely a common consequence of *Setd1a* heterozygosity across different brain regions and cell types, despite the underlying transcriptional alternations are largely neuron (sub)type specific.

### Neuron subtype– and region-specific transcriptional changes in *Setd1a*^+/−^ mouse striatum

Recent single-cell transcriptome profiling has uncovered the neuronal heterogeneity beyond the conventional D1/D2 MSN in striatum, which may underlie the structural/functional complexity of this brain region (*25*). To determine whether *Setd1a* heterozygosity causes uniform or neuron subtype–specific transcriptional changes on the striatal MSN subtypes, we classified the original D1 and D2 MSN populations into five D1 and six D2 subtypes based on their gene expression profiles (Fig. 2, G and H, and fig. S2, E and F). Analysis of the D1 and D2 MSN subtype–specific marker gene expression patterns indicated that these MSN subtypes exhibit distinct spatial distribution in striatum. For example, the D1 MSN subtype 4 and D2 MSN subtype 4 show high expression of *Rasgrf2* (fig. S2, E and F), which are located in the dorsolateral part of the striatum (fig. S2G). On the other hand, the D1 MSN subtype 2 and D2 MSN subtype 6 show high level of *Stard5* and *Upb1* (fig. S2, E and F) and are mainly located in the ventral medial striatum (fig. S2G).

Although *Setd1a* heterozygosity does not affect the relative abundance of the different D1 and D2 subtypes (fig. S2, H and I), it does have different transcriptional effects on different D1 or D2 subtypes with DEGs ranging from a handful to more than 300 (Fig. 2, I and J, and tables S6 and S7). Specifically, the D1 MSN subtype 4 and D2 MSN subtype 4 exhibit the most transcriptional changes, with more than 300 DEGs in each of the two subtypes (Fig. 2, I and J). In addition, around 100 DEGs were identified in D1 MSN subtype 2 and D2 MSN subtype 6, while the other MSN subtypes only showed mild changes in gene expression (DEGs < 25) in the *Setd1a*^+/−^ mice (Fig. 2, I and J). Only a small number of DEGs are shared between different D1 and D2 MSN subtypes (fig. S2, J and K). Since different MSN subtypes could be assigned to different striatal regions (fig. S2G), these results suggested that *Setd1a* deficiency caused different molecular changes in striatal subregions, with the dorsal striatum more severely affected than the ventral striatum (Fig. 2, I and J).

To gain insight into how the gene expression changes might affect neural function in different MSN subtypes, we performed GO analysis with the DEGs in different D1 and D2 MSN subtypes. The results showed that different biological pathways were affected in different MSN subtypes. Although the DEGs of D1 MSN subtype 4 and D2 MSN subtype 4 are both enriched for some common terms (such as mitochondrial translation and regulation of exocytosis), most of their enriched terms are different, such as regulation of ion homeostasis and protein kinase activity in D1 MSN subtype 4, as well as regulation of synaptic vesicle endocytosis and priming in D2 MSN subtypes (Fig. 2K and fig. S2L). This result indicates that *Setd1a* heterozygosity has different transcriptional effects on different MSN subtypes. Collectively, our results suggest that Setd1a deficiency causes distinct transcriptional changes in different MSN subtypes and striatal subregions, with the dorsolateral striatum exhibiting more prominent changes than the ventromedial striatum.

### *Setd1a* heterozygosity does not affect global H3K4me3 pattern in PFC

**Fig. 3.**
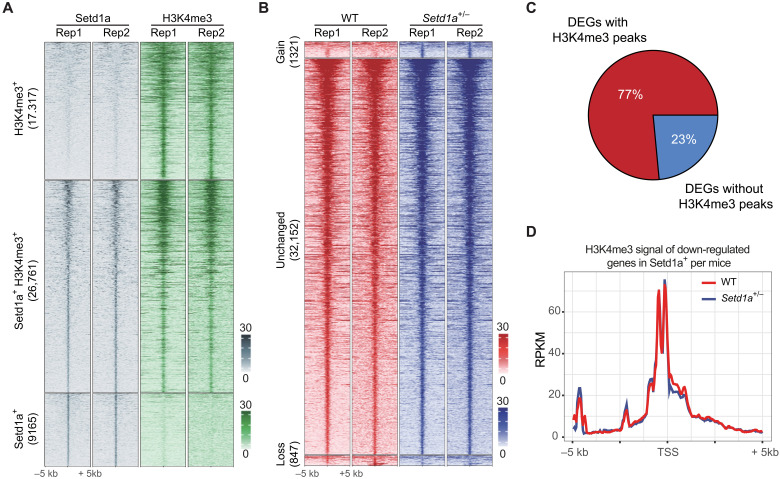
*Setd1a* heterozygosity only causes mild H3K4me3 alterations in the whole PFC. (**A**) Heatmap showing the Setd1a and H3K4me3 peaks detected by CUT&RUN in whole PFC tissue. The Setd1a peaks were classified on the basis of their overlap with H3K4me3 peaks and were sorted in each category on the basis of their signal intensity. The RPKM (reads per kilobase per million mapped reads) intensity of the CUT&RUN peaks is color-coded. (**B**) Heatmap showing the H3K4me3 peaks detected by CUT&RUN in PFC tissues of WT and *Setd1a*^+/−^ mice. The peaks are classified as gain (signal increase in *Setd1a*^+/−^mice) and unchanged and loss (signal decrease in *Setd1a*^+/−^ mice) groups and sorted by their signal intensity. The RPKM intensity of the H3K4me3 signal is color-coded. (**C**) Pie chart showing the percentage of DEGs in PFC (identified by scRNA-seq) with or without associated H3K4me3 peaks. (**D**) Average H3K4me3 signal in genes down-regulated in PFC of *Setd1a*^+/−^ mice (identified by scRNA-seq). DEGs from different cell types were pooled together. The average H3K4me3 RPKM signal between −5 and +5 kb of transcription start sites (TSSs) of WT and *Setd1a*^+/−^ mice is shown in red and blue, respectively.

Setd1a has been previously shown to be an H3K4 methyltransferase (*17*). To gain insight into how *Setd1a* heterozygosity affects transcription in PFC, we perform CUT&RUN assays (*26*) to examine the genomic location of Setd1a protein and H3K4me3 distribution in PFC of WT and *Setd1a*^+/−^ mice (Fig. 3A and fig. S3A). In total, we identified more than 35,000 Setd1a binding sites and 44,000 H3K4me3 peaks. About 50% of the H3K4me3 peaks are located in the promoter region (fig. S3B), consistent with previous studies demonstrating that H3K4me3 is mainly located in the promoter region (*27*). Compared to the H3K4me3 peaks, Setd1a peaks are less enriched in the promoter region (~40%) but are slightly biased to the intronic and intergenic regions (fig. S3B). In general, the peaks of Setd1a and H3K4me3 have good correlation as about 60% of the H3K4me3 peaks overlapped with Setd1a peaks (fig. S3C). In addition, compared to the H3K4me3 peaks without Setd1a binding, the H3K4me3 peaks with Setd1a binding are significantly stronger (fig. S3D). These results indicate that Setd1a is likely involved in H3K4me3 deposition in the PFC.

To examine whether *Setd1a* heterozygosity affects genomic distribution of H3K4me3, we compared H3K4me3 profiles in PFC of WT and *Setd1a*^+/−^ mice. Unexpectedly, only a very small number of H3K4me3 peaks were affected by *Setd1a* heterozygosity (FC > 2 and FDR < 0.05) (Fig. 3B). Furthermore, although most DEGs identified by scRNA-seq of PFC samples have promoter H3K4me3 peaks (Fig. 3C), the H3K4me3 level in these peaks showed little changes in *Setd1a*^+/−^ mice (Fig. 3D and fig. S3E). Thus, the transcriptional changes detected in *Setd1a*^+/−^ PFC by scRNA-seq cannot be directly correlated to the H3K4me3 changes when all the cells in PFC are analyzed as a mixture. One possible explanation is that the H3K4me3 change mainly occurred in specific cell types in PFC, which is masked when the analysis was performed using the entire PFC.

### H3K4me3 decrease is associated with transcriptional decrease in the *Foxp2*^+^ neurons of *Setd1a*^+/−^ mouse PFC

**Fig. 4.**
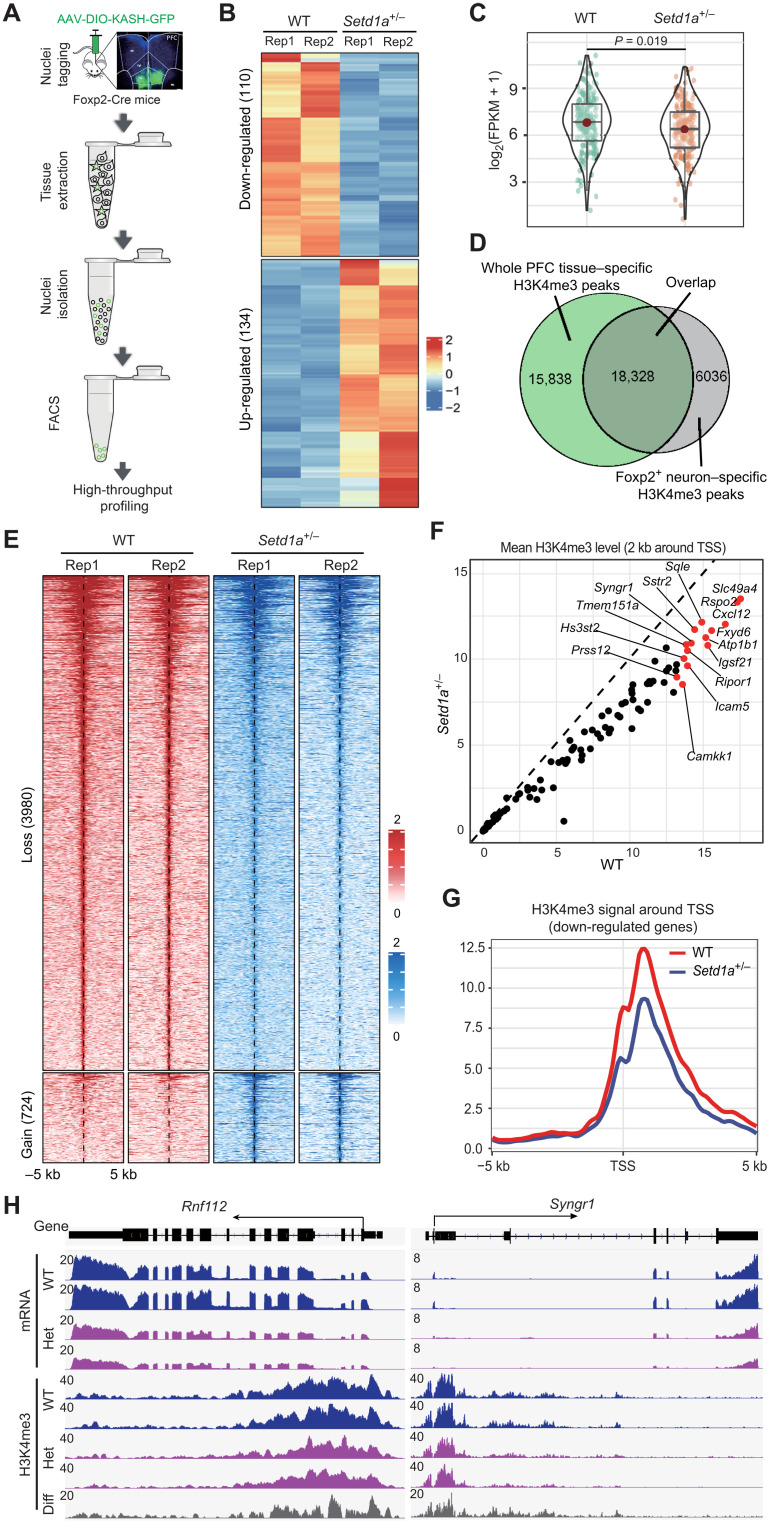
*Setd1a* heterozygosity–caused cell type–specific H3K4me3 changes correlate with transcriptional changes. (**A**) Experimental scheme for cell type–specific transcriptional and epigenetic profiling of PFC *Foxp2*^+^ neurons. (**B**) Heatmap showing the down- and up-regulated genes in *Foxp2*^+^ neurons in *Setd1a*^+/−^ mice. The gene expression level is color-coded. (**C**) Violin plot showing the expression level of *Foxp2*^+^ neuron–specific down-regulated genes (identified by scRNA-seq) in WT and *Setd1a*^+/−^ mice. FPKM, fragments per kilobase per million fragments mapped. (**D**) The overlap of H3K4me3 peaks detected by CUT&RUN in whole PFC tissue and *Foxp2*^+^ neurons. (**E**) Heatmap showing the H3K4me3 peaks with significant changes in *Foxp2*^+^ neurons of *Setd1a*^+/−^ mice. The H3K4me3 signal is color-coded. (**F**) Scatter plot showing the H3K4me3 signal around the TSSs in *Foxp2*^+^ cortical neurons. Only genes showing significantly decreased transcription in *Setd1a*^+/−^ mice were plotted. Red dots represent genes with most significant H3K4me3 decrease. (**G**) Average H3K4me3 signal around the transcriptional start sites of genes down-regulated in PFC *Foxp2*^+^ neurons of *Setd1a*^+/−^ mice (identified by *Foxp2*^+^ neuron–specific RNA-seq). (**H**) Genome browser view showing decreased gene expression and H3K4me3 in *Foxp2*^+^ neurons of *Setd1a*^+/−^ mice. The “Diff” track represents the difference of H3K4me3 signal between WT and *Setd1a*^+/−^ samples (WT minus *Setd1a*^+/−^).

Because scRNA-seq showed that transcriptional changes in the PFC of *Setd1a*^+/−^ mice exhibit cell type specificity, we hypothesized that H3K4me3 changes in PFC of *Setd1a*^+/−^ mice might also exhibit a similar cell type specificity. To test for this possibility, we focused our effort on the DL-Foxp2 neurons, an excitatory neuron subtype in PFC that exhibits the most transcriptional changes in response to *Setd1a* heterozygosity (Fig. 1G). To this end, we crossed the *Foxp2*^Cre^ mice with *Setd1a*^+/−^ mice to generate *Foxp2*^Cre^ /*Setd1a*^+/−^ mice and corresponding control littermates (*Foxp2*^Cre^ /*Setd1a*^+/+^) and then injected a Cre-dependent KASH (Klarsicht, ANC-1, Syne Homology)–green fluorescent protein (GFP)–expressing adeno-associated virus (AAV) into the PFC of these mice so that the nuclei of the *Foxp2*-expressing excitatory neurons in PFC can be tagged by KASH-GFP. By collecting PFC tissues from the injected mice and performing nuclei purification and fluorescence-activated cell sorting (FACS) as described previously (*28*), we obtained nuclei from *Foxp2*^+^ neurons (GFP^+^) for gene expression and H3K4me3 profiling (Fig. 4A).

RNA-seq analysis of *Foxp2*+ excitatory neurons identified 110 down-regulated genes and 134 up-regulated genes in *Setd1a*^+/−^ mice, comparing to WT mice (Fig. 4B and table S5). To compare the *Foxp2*^+^ neuron–specific RNA-seq results with the results obtained from scRNA-seq, we focused on the down-regulated genes, which are more likely to be the direct targets of Setd1a. The results showed that most down-regulated genes in the DL-Foxp2 subtype identified by scRNA-seq also tend to decrease in the RNA-seq of the *Foxp2*^+^ neurons of the *Setd1a*^+/−^ mouse PFC (fig. S4A). As a group, they are significantly down-regulated in *Setd1a*^+/−^ mice compared to that in the control WT mice (Fig. 4C), indicating that the results are consistent when different approaches are used. Consistent with our findings described above, a notable number of DEGs identified from *Foxp2*^+^ neurons have been implicated in SCZ pathogenesis, including *Bcan*, *Bsg*, *Igf2*, *Nptx2*, and *Syngr1*. Furthermore, many of the DEGs are involved in regulating neural morphogenesis and function. For example, *Amigo2* and *Pdlim5* are involved in neuronal morphogenesis, while *Efnb3*, *Icam5*, *Rnf112*, and *Tspan7* are involved in synapse development. In addition, *Aplp1*, *Slc17a7*, and *Syngr1* have been found to play an important role in exocytosis function. These results confirm the vulnerability of the *Foxp2*^+^ neurons upon Setd1a heterozygosity and suggest that the impaired structure and function of these cells may contribute to SCZ-related dysfunctions of the *Setd1a*^+/−^ mice.

To assess the relationship between transcription and H3K4me3 within a relatively homogeneous cell population, we performed parallel CUT&RUN assay to analyze the H3K4me3 profiles with sorted GFP^+^ nuclei from *Foxp2*^Cre^/*Setd1a*^+/+^ and *Foxp2*^Cre^/*Setd1a*^+/−^ mice. Compared to the H3K4me3 profiles generated using the whole PFC tissue with that of the sorted DL-Foxp2 subtype, we identified both shared and differential H3K4me3 peaks (Fig. 4D). Specifically, we found that similar H3K4me3 peaks are present in the promoter regions of genes broadly expressed in PFC (such as *Snap25*) and specifically expressed in DL-Foxp2 neurons (such as *Foxp2*) (fig. S4B). On the other hand, the H3K4me3 peaks in the DL-Foxp2 neurons were absent or significantly lower in the promoter regions of genes not expressed in the *Foxp2*^+^ neurons, such as *Cux2* (marker of superficial cortical layer neurons), *Gja1* (marker of astrocyte), *Mobp* (oligodendrocyte), and *Pdgfra* (oligodendrocyte progenitor cell) (fig. S4B). These results validate the specificity of our cell type–specific H3K4me3 profiling experiments.

We next analyzed the effect of Setd1a heterozygosity on the H3K4me3 pattern in the DL-Foxp2 subtype. With a cutoff of FC > 2 and FDR < 0.05, we identified 3980 sites with decreased and 724 sites with increased H3K4me3 signals in the *Setd1a*^+/−^ mice (Fig. 4E). Most of these affected genomic sites are located in the promoter and gene body (fig. S4C), indicating that they may be involved in transcriptional regulation. Notably, that number of regions that exhibited H3K4me3 loss is more than five times of that gaining H3K4me3 upon *Setd1a* deficiency (Fig. 4E), and the sites with H3K4me3 loss have much higher basal H3K4me3 level compared to that of the sites with H3K4me3 gain (fig. S4D), indicating that

*Setd1a* deficiency mainly causes H3K4me3 loss in DL-Foxp2 neurons, consistent with its established H3K4me3 methyltransferase activity. We next analyzed the relationship between H3K4me3 change and gene expression change and found that most down-regulated genes also showed significantly decreased H3K4me3 peaks (FDR < 0.05) in the *Foxp2*^+^ neuron of the *Setd1a*^+/−^ mice (Fig. 4F). In addition, the overall H3K4me3 signal around the transcription start sites (TSSs) of the down-regulated genes was significantly decreased in the *Setd1a*^+/−^ mice (Fig. 4G), indicating that *Setd1a* deficiency-induced H3K4me3 decrease is likely responsible for the transcriptional dysregulation in the *Foxp2*^+^ neurons of the *Setd1a*^+/−^ mice. For example, *Rnf112* and *Syngr1*, two genes playing important roles in neural function and being associated with human SCZ (*29*, *30*), are significantly down-regulated in *Setd1a*^+/−^ mice and exhibited decreased H3K4me3 signal in their promoter regions (Fig. 4H). Collectively, these results support the notion that *Setd1a* heterozygosity–induced H3K4me3 decrease underlies the transcriptional dysregulation in cortical *Foxp2*^+^ neurons of *Setd1a*^+/−^ mice, and this effect is largely cell type specific, highlighting the advantage of applying cell type–specific approach to uncovering the function of SCZ-associated genes.

### *Setd1a* heterozygosity causes impaired exocytotic function in PFC and striatum neurons

**Fig. 5.**
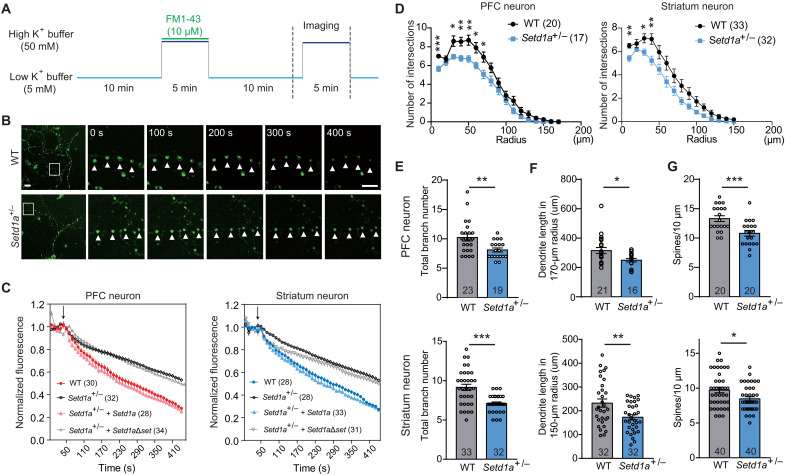
*Setd1a* heterozygosity causes morphological and functional abnormalities in PFC and striatal neurons. (**A**) Experimental scheme of time-lapse imaging analysis of exocytosis. (**B**) Representative images of K^+^-induced exocytosis. The boxed regions were enlarged and shown on the right at indicated time points. Arrowheads indicate vesicles labeled by FM1-43. Scale bars, 10 μm (left) and 5 μm (right). (**C**) Time-lapse recording of K^+^-induced exocytosis in cortical (left) and striatal (right) neurons. The FM1-43 signals at different time points were plotted. WT, neurons from WT mice; *Setd1a*^+/−^, neurons from *Setd1a*^+/−^ mice; *Setd1a*^+/−^ + *Setd1a*, neurons from *Setd1a*^+/−^ mice transfected with full-length Setd1a; *Setd1a*^+/−^ + *Setd1a*Δ*set*, neurons from *Setd1a*^+/−^ mice transfected with Setd1a lacking the Set domain. The arrow indicates the time when 50 mM K^+^ was applied. (**D**) Sholl analysis of PFC (left) and striatal (right) neurons of WT and *Setd1a*^+/−^ mice. (**E** and **F**) Bar graphs showing the total dendrite number (E) and dendrite length (F) of PFC (top) and striatal (bottom) neurons of WT and *Setd1a*^+/−^ mice. (**G**) Bar graphs showing the spine density of PFC (top) and striatal (bottom) neurons in the WT and *Setd1a*^+/−^ mice. Data are shown as means ± SEM. Each dot represents data from an individual neuron. *P < 0.05, **P < 0.01, and ***P < 0.001.

The studies described above reveal transcriptional maladaptation of genes associated with presynaptic functions in PFC (e.g., *Syt10*, *Syp*, *Stx1b*, and *Clcn3*) and striatum (e.g., *Borcs5*, *Ap3m1*, *Ap3s2*, and *Rab26*), raising the possibility that presynaptic function in these brain regions might be impaired in the *Setd1a*^+/−^ mice. To test this possibility, we analyzed exocytosis of synaptic vesicles of cultured neurons from PFC and striatum of WT and *Setd1a*^+/−^animals. Specifically, we isolated cortical and striatal neurons from newborn [postnatal day 0 (P0)] mice and cultured in vitro for 18 days (DIV18) and then performed live imaging to compare the dynamics of K^+^-induced vesicle exocytosis in WT and *Setd1a*^+/−^ neurons (Fig. 5A). We found that compared to the control group, K^+^-induced decrease in fluorescent signal (which label synaptic vesicles) is significantly slower in the *Setd1a*^+/−^ group, in both cortical and striatal neurons (Fig. 5, B and C), indicating that *Setd1a* heterozygosity impaired presynaptic exocytosis. To further test whether this defect was related to the Setd1a catalytic deficiency, we performed rescue experiment by transfecting *Setd1a*^+/−^ neurons with either the full-length Setd1a construct (*Setd1a*) or the *Setd1a* construct lacking the catalytic SET domain (*Setd1a*Δ*set*) at DIV10 and then examined synaptic vesicle exocytosis at DIV18. The results showed that transfecting WT *Setd1a*, but not the catalytic mutant, restored the exocytosis in *Setd1a*^+/−^ neurons to a level comparable to that of the WT neurons (Fig. 5C). These results demonstrate that *Setd1a* heterozygosity causes impairment of exocytotic function in cortical pyramidal neurons and striatal MSNs, and the impairment is related to the defect in the H3K4me3 methyltransferase activity of Setd1a.

### *Setd1a* heterozygosity causes morphological defects in PFC and striatum neurons

Morphological changes in dendrite and spine structure have been observed in both patients with SCZ and mouse models harboring SCZ-associated mutations (*31*–*33*). Consistently, we found that *Setd1a* deficiency led to transcriptional maladaptation of genes involved in neural morphogenesis in both PFC (e.g., *Dnm1l*, *Taok2*, and *Cbln4*) and striatum (e.g., *L1cam*, *Map6*, and *Sdccag8*). To directly assess whether neuronal morphogenesis was impaired in *Setd1a*^+/−^ mice, we focused on DL pyramidal neurons in PFC and MSNs in striatum, as marked transcriptional changes were found in these cells (Figs. 1G and 2C). By performing Golgi staining in brain slices, we compared the dendrite and spine morphology of sparsely labeled neurons between *Setd1a*^+/−^ mice and their WT littermates. The sholl analysis showed that Setd1a heterozygosity decreased dendrite tree complexity in both PFC and striatum (Fig. 5D). Consistently, the branch number (Fig. 5E) and the total dendritic length (Fig. 5F) of the PFC pyramidal neurons and striatal MSNs are significantly deceased in the *Setd1a*^+/−^ mice. In addition, the spine density was also significant decreased in the *Setd1a*^+/−^ mice (Fig. 5G). Collectively, these results indicate that *Setd1a* heterozygosity causes morphological defects in PFC and striatum neurons.

### *Setd1a*^+/−^ mice exhibit a sensorimotor gating defect associated with SCZ

**Fig. 6.**
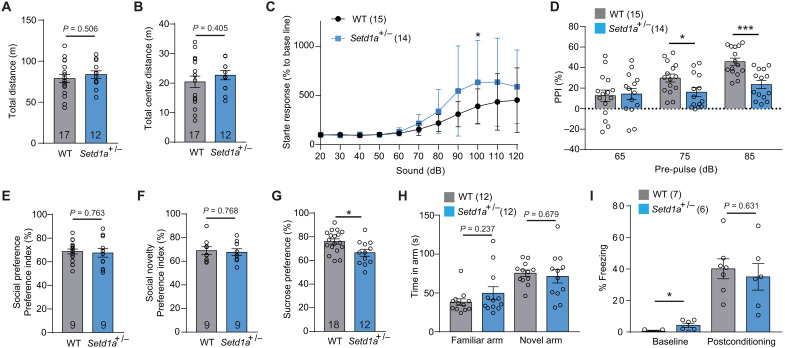
*Setd1a*^+/−^ mice exhibit behavioral defect associated with SCZ. (**A** and **B**) Bar graphs showing the total (A) and central traveling distance (B) in open-field tests of WT and *Setd1a*^+/−^ mice. (**C**) Average startle response of WT and *Setd1a*^+/−^ mice to sound stimulation at different intensities. (**D**) Bar graph showing the PPI values of WT and *Setd1a*^+/−^ mice at three different prepulse intensities (65, 75, and 85 dB). (**E** and **F**) Three-chamber test revealed no significant difference of social preference (E) and social novelty (F) between WT and *Setd1a*^+/−^ mice. (**G**) Sucrose preference test revealed no significant difference between WT and *Setd1a*^+/−^ mice. (**H**) Y-maze test revealed no significant difference between WT and *Setd1a*^+/−^ mice. The time spent in familiar arm and novel arm was plotted. (**I**) Fear conditioning test revealed no significant difference between WT and *Setd1a*^+/−^ mice. The percentage of freezing time before (baseline) and after (postconditioning) conditioning was plotted. Data were presented as means ± SEM. Each dot represents the result from an individual mouse. *P < 0.05 and ***P < 0.001.

Given the molecular and cellular defects observed in *Setd1a*^+/−^ PFC and striatum neurons, we next asked whether these defects can cause behavior changes that mimic certain behavioral abnormality associated with SCZ. To this end, we performed a series of behavioral tests with WT and *Setd1a*^+/−^ mice. For positive symptom–related behaviors, we first used open-field test to assess locomotor activity, which revealed no significant difference between *Setd1a*^+/−^ mice and WT littermates (Fig. 6, A and B). In addition, we analyzed the startle response and prepulse inhibition (PPI) to assess sensorimotor gating. We found that *Setd1a*^+/−^ mice exhibited a slightly larger (but not statistically significant) startle response compared to WT control animals (Fig. 6C). As expected, the PPI effect were observed in WT mice, as presenting a low-intensity audio stimulation (65/75/85 dB) to WT mice resulted in a significantly decreased response to a subsequent stronger audio stimulation (120 dB) (Fig. 6D). However, this PPI effect was significantly reduced in the *Setd1a*^+/−^ mice (Fig. 6D), indicating an impairment of sensorimotor gating in the *Setd1a*^+/−^ mice. Since PPI deficiency is believed to be one of the common sign in patients with SCZ (*34*), we conclude that the *Setd1a*^+/−^ mice exhibit the PPI behavioral defect associated with SCZ.

In addition to the positive symptom–related behaviors, we tested negative symptom–related behaviors, including three-chamber test for social behavior and sucrose preference test for anhedonia phenotype. Although no significant difference was observed between WT and mutant mice in social behavior (Fig. 6, E and F), sucrose preference test revealed a slight but significant decrease in *Setd1a*^+/−^ mice compared to WT mice (Fig. 6G). For cognitive symptom–related behavior, we used Y-maze to assess the short-term memory and found no significant difference between *Setd1a*^+/−^and WT mice (Fig. 6H) regarding the time they spent in the familiar arm and the novel arm. In addition, we performed fear conditioning test to assess the long-term memory. Although *Setd1a*^+/−^ mice showed slightly higher freezing level before conditioning (baseline), both *Setd1a*^+/−^ and WT mice exhibited robust freezing behavior in postconditioning test, and no significant difference was observed between the two genotypes (Fig. 6I). These data suggest that short- and long-term memory were unaffected by Setd1a heterozygosity. Collectively, our behavioral tests revealed sensorimotor gating defect and anhedonia in the *Setd1a*^+/−^mice, suggesting that the Setd1a^+/−^ mouse model only partially recapitulate pathophysiological symptoms of human SCZ.

## DISCUSSION

Human genetic studies have linked SCZ pathogenesis to an increased list of SCZ-susceptible genes (*3*–*6*). Understanding how these genetic mutations contribute to the SCZ pathogenesis is essential for developing effective intervention. In this study, we generated a *Setd1a* haploinsufficiency mouse model and characterized its molecular and cellular effects in PFC and striatum, two brain regions heavily involved in SCZ pathology, with the hope of shedding light on how *SETD1A* mutation contributes to SCZ pathogenesis. Our studies have revealed several important points on how Setd1a haploinsufficiency contributes to the molecular and cellular changes that may underlies SCZ pathogenesis. First, by using scRNA-seq in both PFC and striatum, we found that *Setd1a* haploinsufficiency results in highly variable transcriptional changes among different cell types and neuron subtypes (Figs. 1 and 2). The most affected cells in PFC are endothelia cells and excitatory neurons, particularly the *Foxp2*^+^ neurons (Fig. 1), while the most affected cells in striatum are the D1 and D2 MSNs, especially those located in the dorsolateral striatum (Fig. 2). Second, parallel cell type–specific RNA-seq and H3K4me3 profiling revealed that Setd1a haploinsufficiency–caused H3K4me3 decrease correlates with decreased gene expression in a specific excitatory neuron subtype DL-Foxp2 (Fig. 4). Third, *Setd1a* haploinsufficiency results in defects in dendrite morphology and impaired exocytosis of PFC and striatum neurons (Fig. 5), consistent with the transcriptional dysregulation observed in these two brain regions. Last, *Setd1a* haploinsufficiency leads to sensorimotor gating defect (Fig. 6, C and D), which is associated with SCZ (*35*). Collectively, our study revealed a brain region and cell type–dependent function of Setd1a in H3K4me3-mediated transcriptional regulation, suggesting Setd1a haploinsufficiency-mediated transcriptional dysregulation in different brain region/cell types underlying the pathogenesis of SCZ.

In addition to our study, two other laboratories also used independent *Setd1a* haploinsufficiency mouse models to analyze the role of Setd1a in SCZ pathogenesis (*36*, *37*). All the three studies observed morphological defects in PFC neurons, particularly the decreased spine density, in the *Setd1a*^+/−^ mice. In addition, both of the previous studies have confirmed the synaptic defects using electrophysiological recording. However, the three studies observed somewhat different behavior phenotypes, which might be caused by the differences in the selection of targeting regions in *Setd1a*, different ages of mice, and different settings/protocols for behavior testing. Nevertheless, all three works observed SCZ-associated behavioral phenotypes in *Setd1a*^+/−^ mice. In addition to confirming that *Setd1a*^+/−^ mice can be a valid mouse model for understanding SCZ, our study advanced the previous works in multiple ways, including the following: (i) with scRNA-seq, our work revealed distinct transcriptional changes across different cell types and neuronal subtypes in PFC and striatum, consistent with the notion that different cell types have different contributions to SCZ (*8*). The vulnerable cell types/neuron subtypes and their molecular changes identified in our study can inspire further mechanistic studies on the role of *Setd1a* in SCZ pathogenesis. (ii) By focusing on the *Foxp2*^+^ PFC neurons, we provided evidence that hypo-H3K4me3 underlies the transcriptional maladaptation in *Setd1a*^+/−^ mice. Although the *SETD1A* mutations identified in human patients with SCZ suggest an essential role of its enzymatic activity, the causal relationship between gene expression and Setd1a-dependent H3K4me3 has not been directly assessed in the brain context previously. Notably, we found this relationship was notable only when the Foxp2-expressing neuron subtype was analyzed, highlighting the importance of using cell type–specific approaches in dissecting the molecular mechanism of complex psychiatric disorders. (iii) By assessing different neuron subtypes in PFC and striatum, we showed that the largely cell type–specific transcriptional changes caused by *Setd1a* deficiency converged on some common neural functions, especially the synaptic function, which were further confirmed experimentally. These findings provide molecular insights into how *Setd1a* insufficiency causes neural dysfunction across multiple cell types and different brain regions and suggest that impairment of synaptic function is a general consequence of *Sed1a* heterozygosity.

In addition to revealing the complex effects of *Setd1a* haploinsufficiency in the nervous system, our findings also provide clues for further investigating the neural mechanism underlying SCZ pathogenesis. For example, we identified the DL-Foxp2 excitatory neuron subtype as the most severely affected neurons in PFC. Since these neurons send projections to the thalamus and the cortical-thalamic pathway is known to play an important role in sensorimotor gating (*38*, *39*), it would be interesting to analyze whether *Setd1a* deficiency in this specific circuitry underlies the sensorimotor gating defects observed in the *Setd1a*^+/−^ mice. On the other hand, we found that a number of genes involved in regulating neural morphogenesis and synaptic function, as well as SCZ-associated genes identified in human patients, are dysregulated in the *Setd1a*^+/−^ mice in a cell type–specific manner. Although it is difficult to directly link a specific molecular change to a behavioral phenotype, we note that several Setd1a-regulated genes, including *Cck*, *Efnb3*, *Gabra5*, *Gabrb2*, *Igf2*, *Pdlim5*, *Slc17a7*, *Mt3*, and *Stx1b*, whose heterozygous/homozygous loss of function have been shown to exhibit sensorimotor gating defect (*40*–*47*). Understanding the contribution of these genes to the *Setd1a* haploinsufficiency–caused neural dysfunction in relevant brain region/cell type will broaden our knowledge of SCZ pathogenesis. Last, since our work revealed that *Setd1a* insufficiency–caused transcriptional dysregulation was due to a decrease in the H3K4me3 level in the relevant neuron subtypes, it raises the possibility that antagonizing this change in specific cell types may rescue the molecular maladaptation and alleviate the cellular and behavioral defects caused by *Setd1a*^+/−^ haploinsufficiency. Because the pharmacological intervention of SCZ (e.g., antipsychotic medications) often causes adverse side effects (*48*), our work suggested that mechanism-based cell type–specific treatment, such as gene therapy, may be a potential alternative with better efficacy and less side effects for SCZ treatment.

## MATERIALS AND METHODS

### Mice

All experiments were conducted in accordance with the National Institute of Health’s (NIH) *Guide for the Care and Use of Laboratory Animals* and approved by the Institutional Animal Care and Use Committee of Boston Children’s Hospital and Harvard Medical School. The *Setd1a* heterozygous loss-of-function (*Setd1a*^+/−^) mice were generated as described below. The *Foxp2*^Cre^ mice were obtained from the Jackson Laboratory (030541). For molecular profiling, 12- to 14-week-old adult male mice were used. For behavioral assays, 12- to 16-week-old mice were used. The mice were housed in groups (three to five mice per cage) in a 12-hour light/12-hour dark cycle, with food and water ad libitum unless otherwise specified.

### Generation of Setd1a^+/−^ mice

To generate *Setd1a*^+/−^ mice, we designed a single-guide RNA (sgRNA) (ATCACTGTCCATGATGGCTGAGG) targeting the 15th exon of mouse Setd1a gene, right before the coding sequence of the SET domain. BLAST with mouse genome and transcripts showed that there are at least three nucleotides are different from the sgRNA sequence in any region of the mouse genome other than the on-target site. The efficacy of the sgRNA was validated in the Neuro-2A cell line by sequencing the genomic region around the sgRNA target.

The zygotic CRISPR injection was performed as previously described (*49*). Briefly, superovulated oocytes from B6D2F1 (BDF1) female mice (6 to 8 weeks old) (Jackson Laboratory, 100006) were inseminated with spermatozoa collected from the caudal epididymis of BDF1 males (9 to 12 weeks). Cas9 mRNA (100 ng/μl) and sgRNA (50 ng/μl) were injected into the cytoplasm of each fertilized egg using a Piezo impact-driven micromanipulator (PRIME TECH). At ~24 hours after fertilization, two-cell embryos were transferred into oviducts of surrogate ICR (Institute of Cancer Research) strain mothers. The Cas9 mRNA and Setd1a sgRNA were synthesized as previously described (*50*). To genotype the F0 pups, the genomic DNA sequence around the sgRNA target region was amplified and sequenced to identify mutations, from which several different mutations were identified. We selected an 8-bp frameshift mutation in the 15th exon of Setd1a (which causes premature translational termination before the SET domain) for subsequent studies. To avoid potential off-target effect, the founder mice were backcrossed with C57BL/6N mice for at least five generations before analysis.

### Tissue dissection and dissociation

For single-cell dissociation of PFC and striatum, 12- to 14-week-old male Setd1a heterozygous and their WT littermates were used. For both PFC and striatum, we profiled two biological replicates. In each biological replicate, one sample from each of WT mice and mutant mice was used. For each sample (either WT or mutant), PFC or striatum tissues from two mice were pooled for dissociation. Cell dissociation was performed as previously described (*51*) with minor modifications. Briefly, the PFC and striatum were dissected from 1-mm-thick coronal sections. The tissues were digested with papain at 30°C for 30 min (PFC) or 40 min (striatum) with constant agitation. After washing, the tissues were triturated with fire polished glass Pasteur pipettes to generate single-cell suspension. To remove debris, the single-cell suspension was purified with OptiPrep gradient centrifugation (*52*). The purified cells were stained with Trypan Blue, and the live cells were counted. During the entire procedure, the tissues and cells were kept in ice-cold solutions except during the papain digestion.

### Single-cell capture, library preparation, and sequencing

The cells suspension was diluted with Dulbecco’s phosphate-buffered saline (PBS) containing 0.01% bovine serum albumin (BSA) to 300 to 330 cells/μl for single-cell capture. Single cells and barcoded beads were captured into droplets with the 10x Chromium platform (10x Genomics, CA) according to the protocol from the manufacturer (*53*). After cell capture, reverse transcription, cDNA amplification, and sequencing library preparation were perform as described previously (*53*). The libraries were sequenced on Illumina HiSeq 2500 sequencer with pair-end sequencing (Read1, 26 bp; Index, 8 bp; Read2, 98 bp).

### Nuclei isolation and FACS

To collect PFC tissue for molecular profiling, mice (*Setd1a*^+/−^ and their WT littermates and *Foxp2*^Cre^/ *Setd1a*^+/−^ and their *Foxp2*^Cre^/WT littermates) were euthanized by inhalation of CO_2_. Brains were rapidly dissected and briefly rinsed in ice-cold PBS. The brains were then cut into 1-mm coronal sections with a brain matrix, and the PFC was dissected and immediately frozen with dry ice. The PFC tissues were stored at 80°C until further processing.

The nuclei isolated from frozen PFC tissues were described previously (*28*). Briefly, frozen PFC samples were homogenized in 1 ml of ice-cold homogenization buffer [320 mM sucrose, 5 mM CaCl_2_, 3 mM Mg(Ac)_2_, 10 mM tris (pH 7.6), 0.1 mM EDTA, 0.1% NP-40, 0.1 mM phenylmethylsulfonyl fluoride (PMSF), 1 mM β-mercaptoethanol, 1% BSA, and 1:250 RNasin Plus RNase Inhibitor (Promega)] using a 1-ml Dounce homogenizer (Wheaton). After 10 min on ice, the homogenate was filtered with 40-μm cell strainer (Thermo Fisher Scientific) and diluted 1 ml of dilution buffer [50% OptiPrep density gradient medium (Sigma-Aldrich), 5 mM CaCl_2_, 3 mM Mg(Ac)_2_, 10 mM tris (pH 7.6), 0.1 mM PMSF, and 1 mM β-mercaptoethanol]. Lysate (0.5 ml) was loaded on the top of 0.5 ml of 29% iso-osmolar OptiPrep solution (in PBS) in a 1.5-ml centrifuge tube and centrifuged at 6000*g* for 10 min at 4°C. After removing the supernatant, the nuclei were resuspended in wash buffer [2.5 mM MgCl_2_, 1% BSA in PBS, and 1:500 RNasin Plus RNase Inhibitor (Promega)]. For non-neuron subtype–specific CUN&RUN, the extracted nuclei were homogenized with TRIzol (Thermo Fisher Scientific). For *Foxp2*^+^ neuron–specific RNA-seq and CUT&RUN, the GFP^+^ nuclei were directly sorted into TRIzol (for RNA-seq) or CUT&RUN wash buffer (for CUN&RUN) with Sony SH800 sorter.

### RNA isolation, library preparation, and sequencing

The RNA was extracted from TRIzol with the Direct-zol RNA Microprep Kit (Zymo) following the manufacturer’s instruction, and the RNA from each sample was eluted in 10 μl of ribonuclease (RNase)–free water and stored at −80°C until further processing. The Smart-Seq V4 Kit (Takara) was used for preparing RNA-seq libraries. The RNA from 1000 sorted nuclei of each sample was used for cDNA generation following the manufacturer’s protocol with nine polymerase chain reaction (PCR) cycles to amplify the cDNA. After measuring cDNA concentration (Qubit dsDNA HS Assay Kit, Thermo Fisher Scientific) and quantifying control with Bioanalyzer 2100 (Bioanalyzer High Sensitivity DNA Analysis, Agilent), 300 ng of cDNA was used for library preparation with the Nextera XT DNA Library Preparation Kit (Illumina) according to the manufacturer’s instruction. The libraries were sequenced on Illumina NextSeq 550 sequencer with pair-end sequencing (Read1, 76 bp; Read2, 76 bp).

### CUT&RUN

The sorted GFP^+^ nuclei were immediately used for CUT&RUN assay. The Setd1a and H3K4me3 CUT&RUN libraries were prepared as previously described (*54*) with some modifications. Briefly, the FACS-sorted nuclei were collected in a 1.5-ml tube containing 50 μl of wash buffer [20 mM Hepes (pH 7.5), 150 mM NaCl, 0.5 mM spermidine, and 1× protease inhibitor cocktails (Sigma-Aldrich)]. The nuclei were then captured with BioMagPlus Concanavalin A (Polysciences) beads and incubated with a primary antibody for 16 hours (for H3K4me3) or 2 hours (for Setd1a) at 4°C in antibody incubation buffer [20 mM Hepes (pH 7.5), 150 mM NaCl, 0.5 mM spermidine, 1× protease inhibitor cocktails (EDTA-free tablet, Roche), 2 mM EDTA, and 0.005% digitonin (Life Technologies)]. The H3K4me3 antibody from Cell Signaling Technology (9727S; 1:100) and Setd1a antibody from Abcam (Ab70378; 1:100) were used. After unbound antibodies were washed away, protein A–MNase (micrococcal nuclease) (pA-MN; a gift from S. Henikoff) was added at a 1:280 ratio (500 ng/ml) and incubated for 3 hours (for H3K4me3) or 1 hour (for Setd1a) at 4°C. After washing, CaCl_2_ was added to a final concentration of 2 mM to activate pA-MN, incubated for 20 min at 4°C, and then stopped by adding 1/10 volume of 10× STOP buffer [1700 mM NaCl, 100 mM EDTA, 20 mM EGTA, RNase A (250 μg/ml; Invitrogen), and glycogen (250 μg/ml; Invitrogen)]. The protein-DNA complexes were released by incubating at 37°C for 10 min, followed by 16,000*g* centrifugation for 5 min at 4°C. The supernatant was transferred to a new Lo-bound tube, 1/100 volume of 10% SDS, and 1/80 volume of Proteinase K (25 mg/ml; Life technologies) were added and incubated at 55°C for at least 1 hour. DNA was then precipitated by phenol/chloroform/isoamylalcohol (25:24:1), followed by ethanol precipitation with glycogen, and then dissolved in water.

Sequencing libraries were prepared using the NEBNext Ultra II DNA library preparation kit for Illumina (New Engliand Biolabs) according to the manufacturer’s instructions with a few modifications. Briefly, end repair was conducted at 20°C for 30 min, followed by dA-tailing at 65°C for 30 min (for H3K4me3) or at 50°C for 60 min (for Setd1a). After adaptor ligation at 20°C for 30 min (for H3K4me3) or at 4°C overnight (for Setd1a), the DNA fragments were purified by 1.8× volume of SPRIselect beads (Beckman Coulter) (for H3K4me3) or 2.2× volume of SPRIselect beads (for Setd1a), followed by 14 cycles (for H3K4me3) or 16 cycles (for Setd1a) of PCR amplification with NEBNext Ultra II Q5 Master Mix (New Engliand Biolabs). The PCR products were cleaned up with 0.9× volume of SPRIselect beads (for H3K4me3) or 1.6× volume of SPRIselect beads (for H3K4me3). The primer dimers in the Setd1a CUT&RUN libraries were further removed by purifying 150- to 250-bp DNA fragments from a native polyacrylamide gel electrophoresis. The CUT&RUN libraries were quantified using the Qubit dsDNA HS Assay Kit (Agilent), and quality control was performed with the Bioanalyzer High Sensitivity DNA Analysis (Agilent). The libraries were sequenced on the Illumina HiSeq 2500 with paired-end 100-bp reads or NextSeq 550 with pair-end 76-bp reads.

### Behavioral assays

All mice used for behavioral assays were 12- to 16-week-old male/female mice and their littermates. Behavioral experiments were conducted during the light phase of the light/dark cycle, between 9:00 a.m. and 5:00 p.m.

#### Open-field tests

A clear box (a 27.3 cm–by–27.3 cm square base with 20.3-cm-high walls) used for the open-field test, and the center zone was 50% of the total area. Before testing, mice were habituated to the test room for at least 20 min. Mice were placed into the center of the box at the start of the assay. Movement was recorded using a measurement (Med Associates, St. Albans, VT, ENV-510) 1 hour in 5-min bins. In addition to regular parameters related to locomotor activity (such as total travel distance, velocity, ambulatory time, and resting time), time spent and distance traveled in the center area of the testing arena were also recorded and analyzed.

#### Y-maze

Mice were allowed to adapt in a separate room for approximately 30 min before the test. Each mouse was run twice in the Y-maze: first, a 3-min habituation phase and then a 3-min test phase. The delay or intertrial interval (ITI) between the end of the habituation phase and start of the test phase is 2 min. During the habituation phase, one arm (either left or right) is blocked. The start arm always remains the same. At the end of the habituation phase, the mouse was placed back into the holding cage for the 2-min ITI. The blockade was then removed, and the maze was lightly cleaned. Then, the mouse was placed back into the start arm for the test trial and returned to the home cage after the trial is completed. Distance and time traveled in the maze were recorded by Noldus EthoVision XT.

#### Novel object recognition

Each mouse was subjected to 5-day testing. On the first day, all mice were adapted to the empty arena (50 cm in length, 50 cm in width, and 40 cm in height) for 30 min. On the following 3 days, the mice were exposed to the same chamber, which now contain two identical objects for 10 min (trainings 1 to 3). On the fifth day, each mouse was placed back in the arena with the same object and a novel object for an additional 10-min test session (test). The EthoVision computer-assisted video-tracking system was used to record time exploring the objects. Preference for exploring the novel object was determined by calculation of a discrimination ratio (discrimination ratio = *T*_novel_ − *T*_familiar_/*T*_novel_ + *T*_familiar_).

#### Three-chamber social interaction

Each chamber (30 cm by 30 cm by 30 cm) contains dividing walls with an open middle section to allow for access. Both outer chambers contain wire cups. Mice were given free access to the apparatus for 10 min (absent of other mice) to habituate and confirm initial unbiased preference. The time spent in each chamber was recorded, and the time spent in close interaction with the nose point within 2 cm of the enclosure was also recorded (EthoVision XT 14). To test for sociability, mice were placed into the middle chamber of the apparatus with one outer chamber containing one mouse confined in wired cup and the other chamber containing a Lego block. For social novelty preference, mice were again placed into middle chamber with one chamber containing the familiar mouse and the other containing the novel mouse confined in wired cups. The sex of familiar and novel mice introduced for assay in social interactions matched the sex of the test subject: Males were paired with males and females with females. For each phase, the test mice explored the entire arena throughout the 10-min trial. The time spent in interacting with the empty wire, strange 1, and strange 2 mice during the 10-min session was recorded.

#### Sucrose preference test

Mice were kept in their home cages and given 48 hours of adaptation before the baseline measurement was performed with two identical bottles. Then, mice were deprived of water for 24 hours before being presented with two identical bottles: one containing water and the other containing 1% sucrose solution (bottle locations were randomly assigned and flipped at 12 hours to control for preference inside). The amount of liquid consumed from each bottle was measured at the same time for three consecutive days. Anhedonia is determined by sucrose preference ratio.

#### Acoustic startle and PPI

Animals are submitted to sessions consisting of 10 blocks of 11 trials each (110 trials total). Within each block, various white noise acoustic stimuli (20 to 120 dB) are presented in a random order with a variable ITI of mean 15 s (10 to 20 s). The duration of the stimulus is 40 ms. Responses are recorded for 150 ms from startle onset and are sampled every millisecond. The order of stimuli is random. Habituation time is 5 min. No background white noise was applied.

Animals are placed in the PPI chambers for a 5-min session of white noise (70 dB) habituation. After this adaptation period, the test session is automatically started. The session begins with a habituation block of six presentations of the startle stimulus alone, followed by 10 PPI blocks of six different types of trials. Trial types are null (no stimuli), startle (120 dB), startle plus prepulse (65, 75, or 85 dB, 5000-Hz tone) and prepulse alone (85 dB, 5000-Hz tone). Each PPI trial begins with a 50-ms null period during which baseline movements are recorded. There is a subsequent 20-ms period during which prepulse stimuli are presented and responses to the prepulse measured. After further 100 ms, the startle stimuli are presented for 40 ms, and responses are recorded for 140 ms from startle onset. Responses are sampled every millisecond. The ITI is variable with an average of 15 s (range from 10 to 20 s). All PPI enclosures are cleaned with water and 70% ethanol following each test. The percent PPI is calculated as (100 – prepulse + startle/startle × 100) will provide a measure of sensorimotor gating performance.

### Golgi staining

Golgi-Cox impregnation was performed according to the manual of the Hito Golgi-Cox OptimStain Kit (Hitobiotec Inc., USA). Brains of Setd1a^+/−^ and WT mice were immersed in the impregnation solution (solution A mixed with an equal volume of B) for 14 days under the dark conditions at room temperature. The brains were then transferred to solution C for 48 hours at 4°C. Serial coronal sections (150 μm) were prepared using a Leica CM3050S microtome. Sections were washed twice in distilled water and placed in a mixture solution (D with E) for 10 min. After rinsing, sections were dehydrated in gradient concentration ethanol (50, 75, 95, and 100%) and lastly cleared in xylene for 5 min twice.

Images were captured using a Zeiss microscope with 40×/0.75 numerical aperture (NA) or 100×/1.3 NA oil objectives and a sequential acquisition setting of 1024 × 1024 pixels. For spine analysis, each image was a *z*-series projection of ∼10 images each, averaged two times and taken at 0.29-μm-depth intervals. In the brain slice with Golgi staining, layer V neurons located in the PFC and nucleus accumbens neurons were visualized at a 40× objective, and dendritic segments with a bright-field *z* series were acquired at 100× oil objectives using a Axiocam 506 mono digital camera (ZEISS).

To isolate neurons from brain slices with Golgi staining, the images were imported to NIH ImageJ software. Five to seven pyramidal neurons from Layer V of PFC (cingulate 1 area and prelimbic cortex) were randomly chosen from each mouse (~30 neurons per group). Pyramidal neurons were identified by their characteristic triangular soma shape. Briefly, a transparent grid with concentric rings, equivalent to 10-μm spacing, was placed over the drawing, and the number of dendrites intersecting each ring and primary branches were assessed.

Ten-micrometer dendritic segments at ~25 μm from the cell soma of the primary dendrites were analyzed. At least five neurons were randomly chosen per animal for each group. Counts and data analysis were conducted by experimenters that were blinded to the experimental conditions.

### Primary prefrontal and striatal neuronal cultures

PFC and striatum from P0 pups were dissected in Hanks’ balanced salt solution and digested with 0.25% trypsin at 37°C for 20 min and dissociated with a 1-ml pipette. Neurons were plated at the density of 1 × 10^5^/ml into Dulbecco’s modified eagle’s medium mixture F12 (Gibco) containing 10% fetal bovine serum on 35-mm glass bottom-coated dishes (Cellvis, D35-20-1-N) with poly-d-lysine (50 μg/ml; Sigma-Aldrich). Neurons attached to the substrate were incubated with neurobasal-A medium (Gibco) containing B-27 (Gibco) supplements and l-GlutaMAX (Gibco). The cultures were maintained in a humidified atmosphere of 5% CO_2_ at 37°C. After 24 hours in culture, 10 mM cytosine-β-d-arabinofuranoside (Sigma-Aldrich) was added to restrict glial cell growth. Medium was half-changed every 2 days.

### Imaging of FM lipophilic styryl dyes destaining in presynaptic terminals of cultured neurons

Neurons DIV18 on glass bottom dishes were incubated for 10 min in a Ca^2 +^ -free and low-K^+^ buffer [140 mM NaCl, 5 mM KCl, 5 mM NaHCO_3_, 1.2 mM NaH_2_PO_4_, 1 mM MgCl_2_, 10 mM glucose, and 10 mM Hepes (pH 7.4)] and were then incubated for 5 min with 10 μM FM4-64 [Molecular Probes, Invitrogen in high-K^+^ buffer, 10 mM Hepes (pH 7.4), 95 mM NaCl, 50 mM KCl, 1 mM MgCl_2_, 5 mM NaHCO_3_, 1.2 mM NaH_2_PO_4_, 1.33 mM CaCl_2_, and 10 mM glucose)], followed by a 10-min wash with a Ca^2+^-free low-K^+^ buffer to remove the surface-bound dye. The chamber was adapted at the stage of a Zeiss 800 microscope. Neurons were imaged with a 63× 1.4 NA oil objective at 512 by 512 full-frame resolution using a 568-nm argon laser to excite the FM4-64 probe. After a 50-s time-lapse imaging of basal fluorescence acquisition, neurons were depolarized with high-K^+^ buffer and imaged for 400 s at 10-s intervals. Images from presynaptic loaded puncta were selected for measuring fluorescence intensities using region of interest areas of 1.5 μm by 1.5 μm. All measures were carried at room temperature (25°C). Fluorescence intensities of time-series images were analyzed by Fiji.

### CUT&RUN data processing

Raw reads were first preprocessed to remove low quality reads and adaptor sequences using Trim Galore (v0.4.5) (https://github.com/FelixKrueger/TrimGalore) with the parameter “--paired.” The trimmed reads were then aligned to the mm10 genome using Bowtie2 (v2.3.4.3) (*55*) with parameters “‘--no-unal --no-mixed --no-discordant -I 150 -X 2000.” SAMtools (v1.9) (*56*) was used to remove low mapping quality (mapq < 10) and unmapped reads. Only uniquely mapped reads were kept for downstream analysis. THOR (*57*) with trimmed mean of M-values (*58*) was used to generate scaled bigwig files. The bigwigs of the CUT&RUN samples with yeast spiked-in data were scaled on the basis of the number of uniquely mapped reads to the yeast genome normalized to 100,000 reads (sf = 10^5^/[#uniquely mapped spike-in reads]). Bigwig tracks were generated using “bamCoverage” command from deepTools (v3.0.2) (*59*) with parameters “--binSize 20 --extendReads 250 --normalizeUsing RPKM --outFileFormat bigwig --minMappingQuality 30 –scaleFactor.” The differential peaks were detected using the R/Bioconductor package Linnorm (v.2.16.0) (*60*) for non–spike-in CUT&RUN data, and we used the R/Bioconductor package csaw (v1.20.0) (*61*) for comparison between spiked-in samples.

### Bulk RNA-seq data processing

Raw reads were first preprocessed to remove low quality reads and adaptor sequences using Trim Galore (v0.4.5) (https://github.com/FelixKrueger/TrimGalore) with the parameter “--paired.” Reads were mapped to the reference genome with STAR (v2.7.3a) (*62*) software. Gene level quantification was performed using RSEM (v.1.3.0) (*63*). Differential gene expression was performed using the R/Bioconductor package edgeR (3.28.0) (*64*).

### scRNA-seq data processing

We used CellRange (v3.0.2) (*53*) to process the libraries generated by the 10x Genomics Single-Cell Chromium Controller. We used the “mkfastq” command to generate demultiplexed FASTQ files from Illumina base call files. Next, the CellRanger command “count” was used to quantify gene expression, identify valid cell barcodes, and generate the initial gene × cell matrix for each sample. We used the R package Seurat (v3.2.0) (*65*) for downstream analysis. We kept all the genes that are expressed in at least three cells and filtered out cells expressing less than 800 genes or having more than 10% of their transcriptome coming from mitochondrial genes. Gene expression in each cell was normalized to 10,000 reads, and the top 2000 variable genes for each sample were identified using function “FindVariableFeatures.” The individual samples of each tissue were combined together using the canonical cross-correlation method from Seurat, by calling the function “FindIntegrationAnchors” followed by the function “IntegrateData” both with parameters “dims = 1:30.” The top 30 principal components were used to generate the uniform manifold approximation and projection (UMAP) dimension reduction representations. Clusters were identified using the function “FindClusters” with a resolution of 0.4. Similar clusters were merged after manual inspection. The R/Bioconductor package DEsingle (v1.12.0) was used to detected DEGs (FDR < 0.05 and FC > 1.5) in each cell type between WT and Setd1a heterozygous cells.

### GO and IPA analysis

GO analysis was performed using the enrichGO functionF R/Bioconductor package clusterProfiler (v3.16.0) (*66*) with the following parameters: ont = “BP,” keyType = “SYMBOL,” minGSSize = 3, OrgDB = “org.Mm.eg.db,” all the genes listed in the org.Mm.eg.db (v3.11) R/Bioconductor package were used as background.

The IPA was performed using the “IPA Core analysis” module by specifying the log_2_FC column as the “Exp Log Ration” and the adjusted *P* value column as “Exp False Discovery rate.” The analysis was performed with default parameters. The analysis was conducted under “canonical pathways” and “disease and functions” that annotate all GO pathways in IPA. A right-tailed Fisher’s exact test *P* value cutoff of at least 0.05 was applied in reporting any significantly enriched functional pathway.

### Quantification of GWAS overlap significance

#### Bootstrapped overlap analysis

We downloaded a list of GWAS candidate genes and their associated disease from the NHGRI-EBI (National Human Genome Research Institute- European Bioinformatics Institute) GWAS catalog (version 1.0.2) (*67*). We only considered the GWAS genes that have an exonic mutation and have been validated by published studies that pass the NHGRI-EBI GWAS catalog eligibility criteria. Genes reported to be associated with SCZ and bipolar disease were consider. Only genes with a homologous mouse gene were kept. A list of 1245 genes was obtained. To test the significance of the observed number of DEG overlapping with the GWAS genes, we did a bootstrapped analysis test. Briefly, for each cell type, we generated 10,000 random gene sets that had the same size as the number of DEGs in the cell type. Then, we calculated the number of GWAS gene overlap for each random gene set. The distribution of random overlaps was used to estimate the *P* value as follows: *pval_ct_* = *P*(*X* > *observedGWASovp_ct_*), where *observedGWASovp_ct_* is the number of observed overlap for a certain cell type *ct* in our data.

#### MAGMA

We also analyzed the overlap between SCZ-associated genes and DEGs of different cell types in PFC and striatum with MAGMA. Specifically, we used MAGMA (v1.09) and the summary statistics of a recent large SCZ GWAS study with 40,675 cases and 64,643 controls (*68*). By using the same setting as Skene *et al.* (*8*), we annotated single-nucleotide polymorphisms onto their neighboring genes if they fall within [TSS, −10 kb; TES (transcription end site), +1.5 kb] of the gene. Then, gene-level trait association statistics were calculated using MAGMA while accounting for linkage disequilibrium structures obtained from the European panel of 1000 Genomes Project. The analysis was performed with the R package MAGMA_Celltyping (https://github.com/neurogenomics/MAGMA_Celltyping) the same way as in the work of Skene *et al.* (*8*). To generate a comparable gene expression metric, the gene expression in each cell type was dividing into 40 bins, and the gene expression value was replaced by expression quantile number. MAGMA was then used to test for positive association between the binned expressions and the gene-level association. As MAGMA requires gene sets with more than 100 genes, we only tested endothelial cell and excitatory neuron in PFC, and D1 MSN, D2 MSN, and inhibitory neuron in striatum.

### Quantification and statistical analysis

All experimental procedure was conducted blinded. Statistical analyses were performed using GraphPad Prism (version 8.0). Datasets with normal distribution were analyzed for significance using Student’s unpaired two-tailed *t* tests. Statistical significance was set at *P < 0.05, **P < 0.01, and ***P < 0.001. Data are presented as means ± SEM.

## Notes

View/request a protocol for this paper from *Bio-protocol*.
