# Supplementary material for: Cell type–specific mechanism of Setd1a heterozygosity in schizophrenia pathogenesis
Source: Sci Adv. 2022 Mar 4;8(9):eabm1077. doi: 10.1126/sciadv.abm1077 (PMC8896793; doi:10.1126/sciadv.abm1077)
Supplement: Supplementary file 1 — Figs. S1 to S4 [file sciadv.abm1077_sm.pdf]

Science Advances
AAAS

Supplementary Materials for

Cell type–specific mechanism of Setd1a heterozygosity in schizophrenia pathogenesis

Renchao Chen, Yiqiong Liu, Mohamed N. Djekidel, Wenqiang Chen, Aritra Bhattacherjee, Zhiyuan Chen, Ed Scolnick, Yi Zhang*

*Corresponding author. Email: yzhang@genetics.med.harvard.edu

Published 4 March 2022, *Sci. Adv.* **8**, eabm1077 (2022)
DOI: 10.1126/sciadv.abm1077

The PDF file includes:

Figs. S1 to S4
Legends for tables S1 to S7

Other Supplementary Material for this manuscript includes the following:

Tables S1 to S7

Fig. S1.

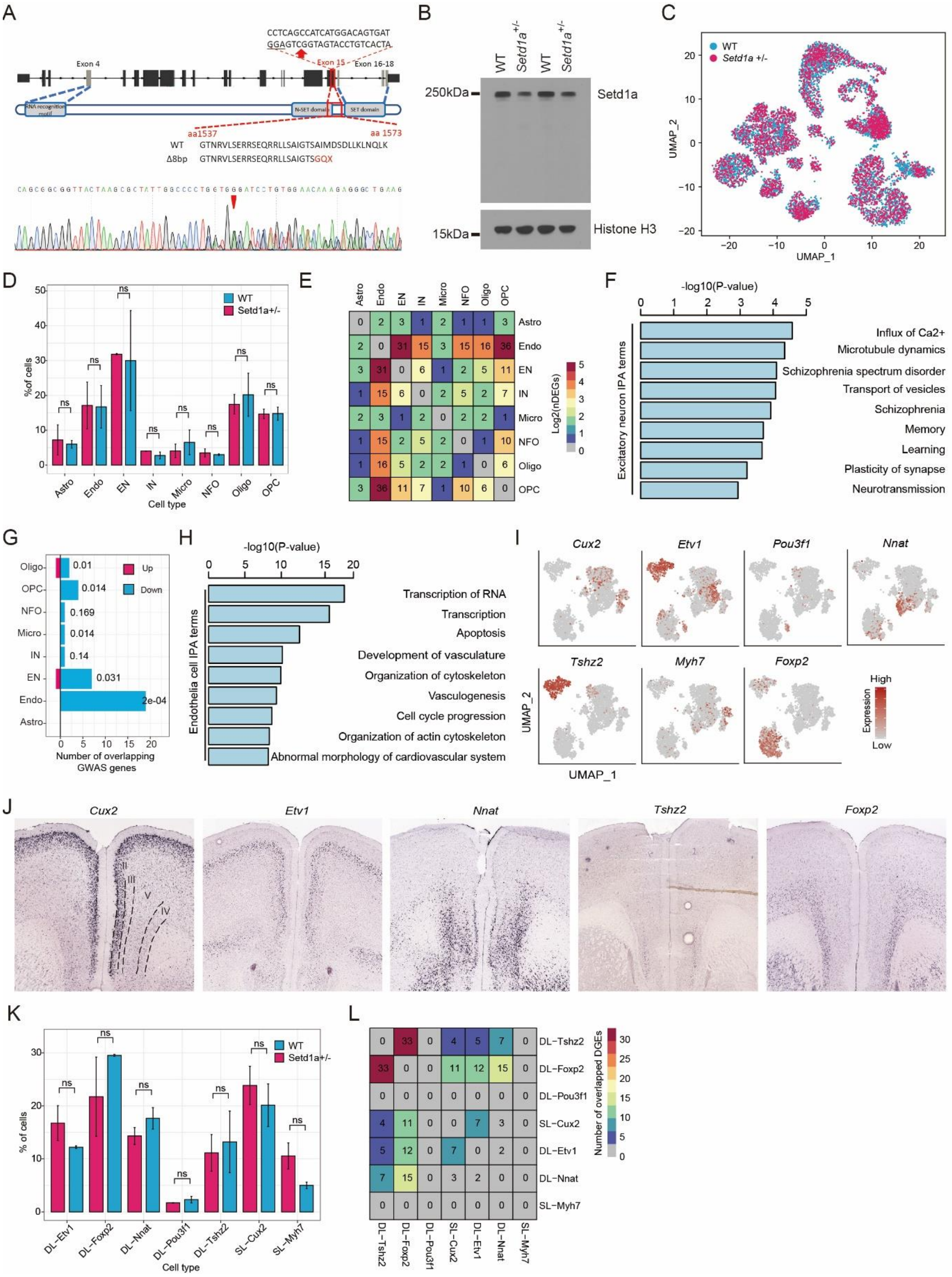

A
CCTCAGCCATCATGGACAGTGAT
GGAGTCGGTAGTACCTGTCACTA
Exon 4
Exon 15
Exon 16-18
RNA recognition motif
N-SET domain
SET domain
aa1537
aa 1573
WT GTNRVLSERRSEQRRLLSAIGTSAIMDSDLLKLNQLK
Δ8bp GTNRVLSERRSEQRRLLSAIGTSGQX
B
WT
Setd1a+/-
250kDa
Setd1a
15kDa
Histone H3
C
WT
Setd1a +/-
UMAP_1
UMAP_2
D
%of cells
Cell type
Setd1a+/-
ns
Astro
Endo
EN
IN
Micro
NFO
Oligo
OPC
E
Log2(nDEGs)
F
-log10(P-value)
Excitatory neuron IPA terms
Influx of Ca2+
Microtubule dynamics
Schizophrenia spectrum disorder
Transport of vesicles
Schizophrenia
Memory
Learning
Plasticity of synapse
Neurotransmission
G
Up
Down
Number of overlapping GWAS genes
H
-log10(P-value)
Endothelia cell IPA terms
Transcription of RNA
Transcription
Apoptosis
Development of vasculature
Organization of cytoskeleton
Vasculogenesis
Cell cycle progression
Organization of actin cytoskeleton
Abnormal morphology of cardiovascular system
I
Cux2
Etv1
Pou3f1
Nnat
Tshz2
Myh7
Foxp2
High
Expression
Low
J
K
% of cells
DL-Etv1
DL-Foxp2
DL-Nnat
DL-Pou3f1
DL-Tshz2
SL-Cux2
SL-Myh7
L
Number of overlapped DGEs

Fig. S1. ***Setd1a*** **heterozygosity causes cell type-specific transcriptional changes in PFC**

(A) Generation of Setd1a loss-of-function mouse model with CRISPR/Cas9. Upper panel, gene structure and protein domains of mouse Setd1a. The CRISPR/Cas9 target site is located in exon 15 before the Set domain coding sequence. The red arrow indicates the cutting site. The premature translational termination caused by an 8bp deletion was illustrated. Lower panel, DNA sequencing confirming the 8bp deletion.

(B) Western blot showing the expression of Setd1a protein in the cortical tissues of wildtype and *Setd1a*$^{+/-}$ mice. Histone H3 was used as a loading control.

(C) UMAP plot showing the cells from the PFC of WT and *Setd1a*$^{+/-}$ mice. Cells from different genotypes were labeled with different colors.

(D) Bar graph showing the percentage of different cell types in PFC of WT and *Setd1a*$^{+/-}$ mice detected by scRNA-seq. Wilcoxon rank test was used to compare the cell proportion between WT and *Setd1a*$^{+/-}$ mice.

(E) Heatmap showing the overlap of differentially expressed genes (DEGs) among different cell types of PFC between WT and *Setd1a*$^{+/-}$ mice identify by scRNA-seq. The number of overlapped DEGs between each pair of cell types was listed, which was also indicated with different colors.

(F) Bar graph showing term enrichment of the differentially expressed genes in the excitatory neurons of PFC from the IPA. The -$\log_{10}$(p-value) of terms is represented by the length of the bar.

(G) Bar graph showing the number of overlapped genes between the DEGs in different cell types of PFC and the schizophrenia-associated genes identified by GWAS studies. P-values were calculated using 10,000 bootstrapped gene sets for each cell type.

(H) Bar graph showing term enrichment of the differentially expressed genes in endothelial cells of PFC from the IPA. The p-value of the terms is represented by the length of the bar.

(I) UMAP plot showing the enrichment of different marker genes in different excitatory neuron subtypes of PFC. The expression level was color-coded.

(J) In situ hybridization images showing the enriched expression of marker genes of different excitatory neurons subtypes in different layers of PFC. Data was obtained from Allen Brain Atlas. Different cortical layers were labeled with dash lines.

(K) Bar graph showing the percentage of different excitatory neurons subtypes in PFC of WT and *Setd1a*$^{+/-}$ mice detected by scRNA-seq. Wilcoxon rank test was used to compare the cell proportion between WT and *Setd1a*$^{+/-}$ mice.

(L) Heatmap showing the overlap of differentially expressed genes (DEGs) among different excitatory neuron subtypes of PFC between WT and *Setd1a*$^{+/-}$ identified by scRNA-seq. The number of overlapped DEGs between each pair of cell types was listed, which was also indicated by different colors.

Fig. S2.

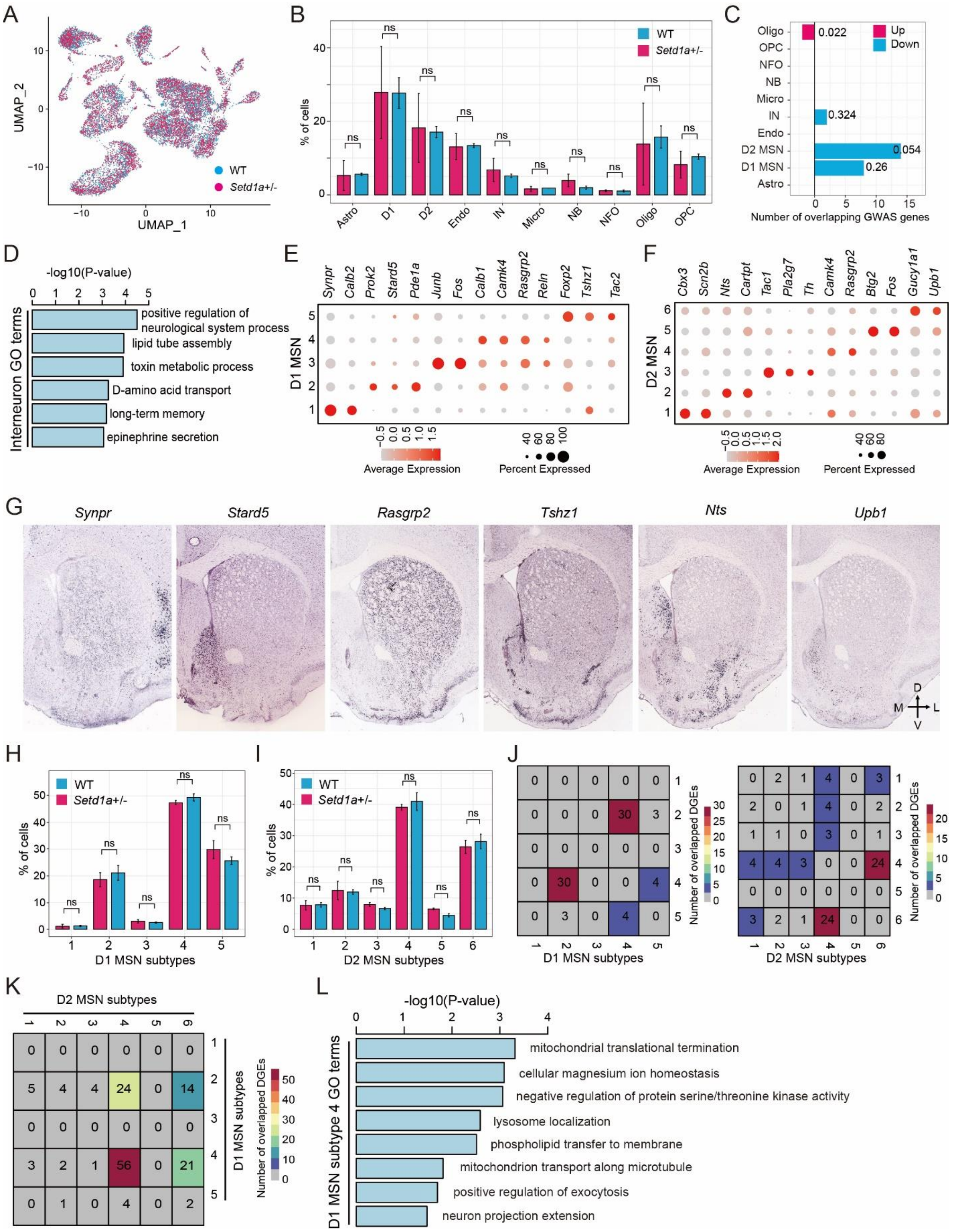

A
UMAP_1
UMAP_2
WT
Setd1a+/-
B
% of cells
ns
WT
Setd1a+/-
Astro
D1
D2
Endo
IN
Micro
NB
NFO
Oligo
OPC
C
Oligo
OPC
NFO
NB
Micro
IN
Endo
D2 MSN
D1 MSN
Astro
0.022
0.324
0.054
0.26
Up
Down
Number of overlapping GWAS genes
D
-log10(P-value)
Interneuron GO terms
positive regulation of neurological system process
lipid tube assembly
toxin metabolic process
D-amino acid transport
long-term memory
epinephrine secretion
E
Synpr
Calb2
Prok2
Stard5
Pde1a
Junb
Fos
Calb1
Camk4
Rasgrp2
Reln
Foxp2
Tshz1
Tac2
D1 MSN
Average Expression
Percent Expressed
F
Cbx3
Scn2b
Nts
Cartpt
Tac1
Pla2g7
Th
Camk4
Rasgrp2
Btg2
Fos
Gucy1a1
Upb1
D2 MSN
Average Expression
Percent Expressed
G
Synpr
Stard5
Rasgrp2
Tshz1
Nts
Upb1
D
M
L
V
H
WT
Setd1a+/-
% of cells
D1 MSN subtypes
I
WT
Setd1a+/-
% of cells
D2 MSN subtypes
J
Number of overlapped DGEs
D1 MSN subtypes
D2 MSN subtypes
K
D2 MSN subtypes
D1 MSN subtypes
Number of overlapped DGEs
L
-log10(P-value)
D1 MSN subtype 4 GO terms
mitochondrial translational termination
cellular magnesium ion homeostasis
negative regulation of protein serine/threonine kinase activity
lysosome localization
phospholipid transfer to membrane
mitochondrion transport along microtubule
positive regulation of exocytosis
neuron projection extension

Fig. S2. ***Setd1a*** **heterozygosity causes cell type-specific transcriptional changes in striatum**

(A) UMAP plot showing the cells from the striatum of WT and *Setd1a*$^{+/-}$ mice. Cells from different genotypes were labeled by different colors.

(B) Bar graph showing the percentage of different cell types in striatum of WT and *Setd1a*$^{+/-}$ mice detected by scRNA-seq. Wilcoxon rank test was used to compare the cell proportion between WT and *Setd1a*$^{+/-}$ mice.

(C) Bar graph showing the overlap between the DEGs in different cell types of striatum and the schizophrenia-associated genes identified by GWAS studies. P-values were calculated using 10,000 bootstrapped gene sets for each cell type.

(D) Bar graph showing GO term enrichment of differentially expressed genes in striatal interneurons. The -$\log_{10}$(p-value) of GO terms is represented by the length of the bar.

(E, F) Dot plots showing the enrichment of different marker genes in different D1 (**E**) and D2 (**F**) MSNs. The size of the dots represents the percentage of cells within an MSN subtype expressing that gene. The gene expression level is color-coded.

(G) *In situ* hybridization images showing the enriched expression of marker genes of different MSN subtypes in different subregions of striatum. The dorsal-ventral (DV) and medial-lateral (ML) axes are indicated. Data was from Allen Brain Atlas.

(H, I) Bar graph showing the percentage of different D1 (**H**) and D2 (**I**) MSN subtypes in striatum of WT and *Setd1a*$^{+/-}$ mice determined by scRNA-seq. Wilcoxon rank test was used to compare the cell proportion between WT and *Setd1a*$^{+/-}$ mice.

(J) Heatmap showing the overlap of differentially expressed genes (DEGs) within different D1 and D2 MSN subtypes between WT and *Setd1a*$^{+/-}$ mice identify by scRNA-seq. The number of overlapped DEGs between each pair of cell types was listed, which was also indicated by different colors.

(K) Heatmap showing the overlap of differentially expressed genes (DEGs) between D1 and D2 MSN subtypes in WT and *Setd1a*$^{+/-}$ mice. The number of overlapped DEGs between each pair of cell types was listed, which was also indicated by different colors.

(L) Bar graph showing GO term enrichment of differentially expressed genes of D1 MSN subtype 4. The length of the bars represent p-value of different GO terms.

Fig. S3.

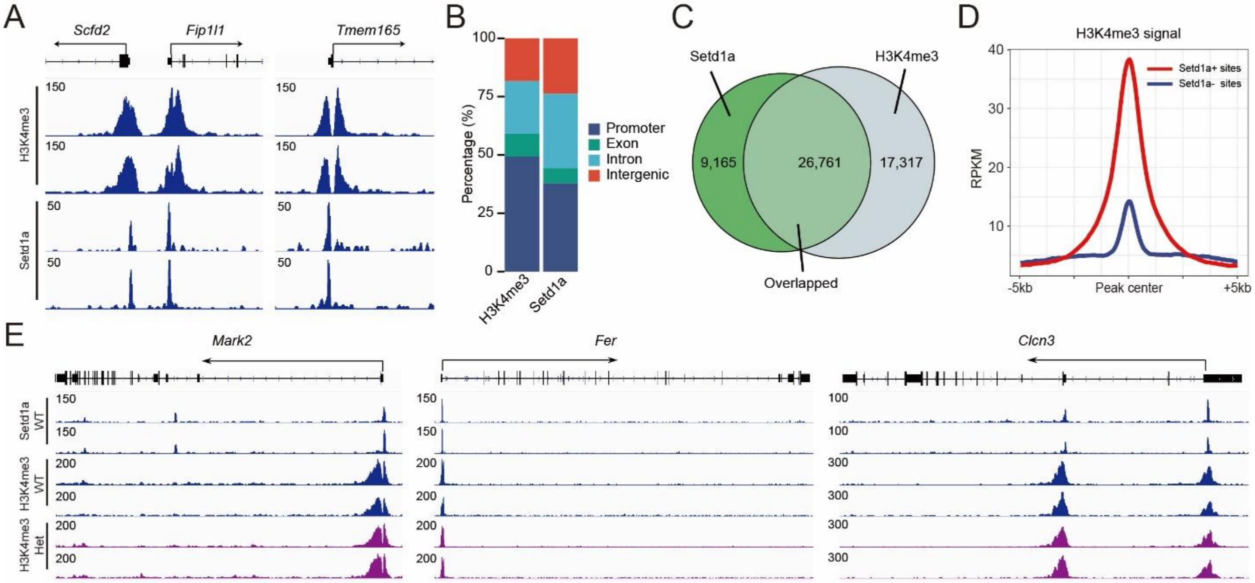

Fig. S3. Sed1a binding and H3K4me3 profiling in PFC

(A) Genome browser view showing the Setd1a and H3K4me3 signals around the *Scfd2*, *Flp1l1* and *Tmem165* locus detected by CUT&RUN assay with corresponding antibodies. Two replicates for each condition were shown.

(B) Bar graph showing the genomic distribution of Setd1a binding sites and H3K4me3 peaks in PFC.

(C) Venn diagram showing the overlap between Setd1a binding sites and H3K4me3 peaks in PFC.

(D) Averaged H3K4me3 RPKM signal in H3K4me3 peaks with (red) or without (blue) corresponding Setd1a binding sites.

(E) Genome browser view of representative loci showing Setd1a binding and H3K4me3 signal in genes with decreased expression in the PFC of *Setd1a*$^{+/-}$ mice identified by scRNA-seq. Two replicates of Setd1a (in WT mice) and H3K4me4 (in both WT and *Setd1a*$^{+/-}$ mice) CUT&RUN were shown. Mark2, Fer and Clcn3 were found to be down-regulated in excitatory neuron, endothelial cell and oligodendrocyte progenitor cell in the PFC of *Setd1a*$^{+/-}$ mice, respectively. The start site and direction of transcription of each gene is indicated.

Fig. S4.

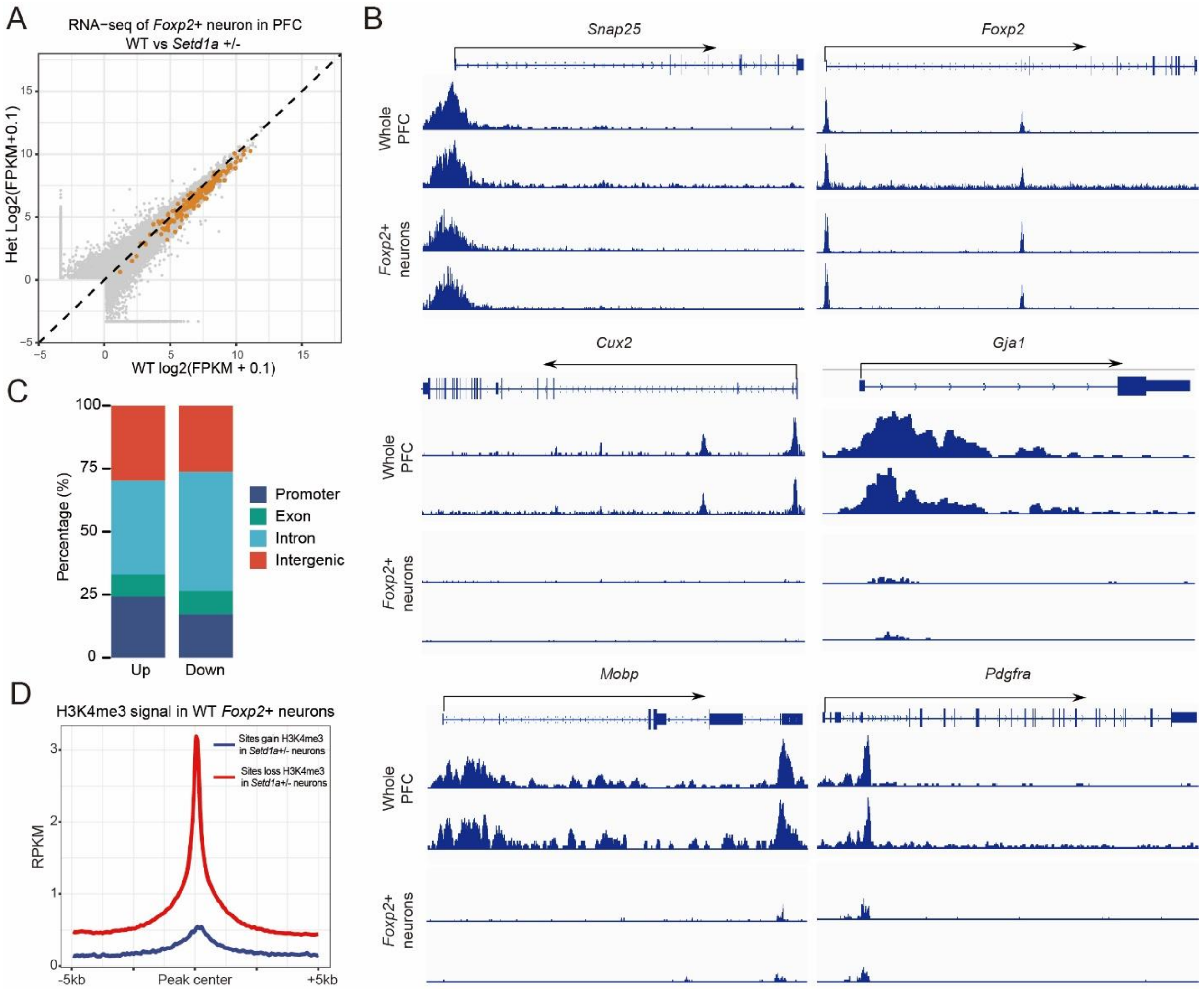

Fig. S4. ***Setd1a*** **heterozygosity causes transcriptional and epigenetic changes in the** ***Foxp2***$^{+}$ **cortical neurons**

(A) Scatter plot showing the expression level of *Foxp2*$^{+}$ neuron specific down-regulated genes (labeled in orange, identified by scRNA-seq) between WT and *Setd1a*$^{+/-}$ mice detected by *Foxp2*$^{+}$ neuron-specific RNA-seq.

(B) Genome browser view showing representative loci with similar (*Snap25*, *Foxp2*) or different (*Cux2*, *Gja1*, *Mobp*, *Pdgfra*) H3K4me3 peaks between whole PFC and *Foxp2*$^{+}$ neurons CUT&RUN assay. Two replicates of each condition were shown. The start site and direction of transcription for each gene is indicated.

(C) Bar graph showing the genomic distribution of H3K4me3 peaks with increased (up) or decreased (down) signal in *Foxp2*$^{+}$ cortical neurons of *Setd1a*$^{+/-}$ mice.

(D) Averaged H3K4me3 RPKM signal of two groups of H3K4me3 peaks in *Foxp2*$^{+}$ neurons of WT mice: H3K4me3 peaks with significantly increased signal in *Setd1a*$^{+/-}$ mice (blue), and H3K4me3 peaks with significantly decreased signal in *Setd1a*$^{+/-}$ mice (red).

Supplementary tables

Table S1. List of differentially expressed genes in different cell types of the *Setd1a*$^{+/-}$ PFC

Table S2. List of overlapped genes between the DEGs in different cell types of the *Setd1a*$^{+/-}$ PFC and schizophrenia associated genes identified by GWAS

Table S3. List of differentially expressed genes in different excitatory neuron subtypes of the *Setd1a*$^{+/-}$ PFC

Table S4. List of differentially expressed genes in different cell types of the *Setd1a*$^{+/-}$ striatum

Table S5. List of differentially expressed genes in the *Foxp2*$^{+}$ neuron subtype of *Setd1a*$^{+/-}$ PFC

Table S6. List of differentially expressed genes in D1 MSN subtypes of *Setd1a*$^{+/-}$ striatum

Table S7. List of differentially expressed genes in D2 MSN subtypes of *Setd1a*$^{+/-}$ striatum
